# *Crotalaria madurensis* flavonol glycosides’ antibacterial activity against *Staphylococcus aureus*

**DOI:** 10.1186/s13568-024-01776-3

**Published:** 2024-11-04

**Authors:** Hala Sh. Mohammed, Salwa A. Abu El Wafa, Mona H. Ibrahim, Rasha Mohammad Fathy, Noha A. Seif-Eldein

**Affiliations:** 1 Pharmacognosy and Medicinal Plants Department, Faculty of Pharmacy, Al-Azhar University, Cairo, Egypt; 2 Department of Pharmaceutical Medicinal Chemistry and Drug Design, Faculty of Pharmacy (Girls), Al-Azhar University, Cairo, 11884 Egypt; 3 Drug Radiation Research Department, National Center for Radiation Research and Technology (NCRRT), Egyptian Atomic Energy Authority, Cairo, Egypt

**Keywords:** Antioxidant, *Crotalaria madurensis*, Flavonol, MRSA, Antibacterial, Docking study

## Abstract

Methicillin-resistant *Staphylococcus aureus* (MRSA) infections are prevalent in hospitals and often lead to significant health complications. This study aimed to explore the chemical composition of the aerial part of *Crotalaria madurensis* and evaluate its antioxidant and antibacterial properties. The impact of gamma irradiation on the antibacterial properties of the plant extract and metabolite 1 against MRSA was also examined. Fourier-transform infrared (FTIR) analysis was conducted on the filtrates of untreated MRSA and MRSA treated with the plant extract and metabolite 1. Four flavonol glycosides were identified as gossypetin 8-methoxy, 3-*O*-β-D-xylopyranoside (metabolite 1), gossypetin 8-*O*-β-D-glucopyranoside (metabolite 2), kaempferol 3-*O*-β-D-glucpyranoside (Astragalin, metabolite 3), and herbacetin 7-methoxy-3-*O*-β-D-glucopyranoside (metabolite 4). All metabolites exhibited significant antioxidant properties using different assays. The antibacterial efficacy of the extract and metabolite 1, which showed substantial antioxidant properties compared to the other isolated metabolites, was evaluated. Both the plant extract and metabolite 1 significantly reduced the viability and cell count of MRSA at concentrations of 1.0 and 0.5 mg/ml. The antibacterial activity of the plant extract and metabolite 1 was assessed after gamma irradiation at 50 and 100 Gy, which did not significantly affect the antibacterial efficiency. FTIR analysis indicated that the plant extract and metabolite 1 significantly altered the band frequency values, bandwidth, and peak intensity % of the treated MRSA filtrate. Molecular docking studies suggested that metabolite 1 exhibited the highest antioxidant and anti-MRSA activity, with strong binding scores like the ligand, indicating an effective interaction and high affinity between metabolite 1 and the target molecule.

**Supplementary Information:**

The online version contains supplementary material available at 10.1186/s13568-024-01776-3.

## Introduction

Antimicrobial resistance is a critical global health issue, necessitating urgent, innovative, and cost-effective solutions. *S. aureus*, a common Gram-positive bacterium, often leads to persistent illness due to its virulence and the formation of dense communities (Andriamampianina et al. 2016; Abalkhail and Elbehiry 2022). Its increasing resistance to vancomycin has led to the emergence of more resistant strains, producing a variety of virulence factors. MRSA, known for its resistance to front-line treatments, develops resistance by transferring van gene clusters, altering the drug’s target (Getahun et al. 2017). The high pathogenicity of MRSA, combined with its resistance to antibiotics, results in life-threatening infections in humans and animals. Given MRSA’s growing resistance, alternative strategies are needed to mitigate losses (Shoaib et al. 2023). Consequently, plant extracts have been systematically screened as potential sources of antibacterial drugs (Stefanovic and Comic 2011). Several Crotalaria species have been reported to possess antimicrobial activity. These species are recognized as a rich source of non-toxic pyrrolizidine alkaloids (PAs) (Asres et al. 2004). In addition to PAs, flavonoid glycosides, prenylated chalcone, dihydrochalcones, chalcones, flavanones, triterpenes, and isoflavones have also been identified (Hala et al. 2008; Khalilullah et al. 1993; Yang et al. 1998). For instance, *C. madurensis* R. Wight (Leguminosae), an ornamental shrub native to the Nilgiris and Madura hills in India, is one of 80 Crotalaria species. *C. madurensis* has demonstrated effectiveness against *Bacillus subtilis*, *Staphylococcus aureus*, *Escherichia coli*, and *Candida albicans* (Pandey and Nayar 1994; Bhakshu et al. 2008). Flavonoids, produced by plants in response to microbial infection, serve as potent antibacterial agents. Their antibacterial properties are largely determined by their structures, specifically the modifications to their aromatic rings. These compounds could target multiple cellular sites and form complexes with proteins through both covalent bonds and non-specific forces. This multi-target approach can potentially enhance their antibacterial efficacy. Their antimicrobial action may be related to their ability to inactivate microbial adhesion, enzymes, and cell membrane transport proteins. Lipophilic flavonoids may also damage microbial membranes (Cowan 1999; Yee and Koo 2000). Several reported studies investigated the antimicrobial effectiveness of 38 flavonoids from seven classes against multidrug-resistant bacteria. Active flavonoids had specific structural requirements. More hydrophilic flavonols or flavones were stronger inhibitors. Tetrahydroxyflavanones had the highest action against MRSA. The study also highlighted the importance of the hydroxyl group at position C-3 and the position of free hydroxyl groups for a molecule’s activity (Xu and Lee 2001; Tsuchiya et al. 1996). Flavonoids, recognized for their antibacterial, anticancer, antiviral, antimutagenic, and anti-inflammatory attributes, serve as powerful antioxidants and free radical scavengers. These compounds, renowned for their antioxidant capabilities, can directly influence bacterial growth and obstruct their pathogenic activities (Tagousop et al. 2018; Mills and Bone 2000; Górniak et al. 2019). They inhibit nucleic acid synthesis, cytoplasmic membrane function, energy metabolism, and altered membrane permeability (Górniak et al. 2019). They can also prevent attachment and biofilm formation, inhibit the cell membrane’s porin, and reduce pathogenicity. Some flavonoids can even counteract antibiotic resistance and boost the effectiveness of existing antibiotic drugs. Thus, the antioxidant activity of flavonoids is intrinsically linked to their antimicrobial activity and could potentially affect antimicrobial resistance. Further research is required to fully comprehend this relationship (Górniak et al. 2019). The goal of the docking study is to predict the mechanism of action based on the interactions between the compounds and the protein's active site. The enzyme arachidonate 5-lipoxygenase (5-LOX) is essential for leukotriene production. According to research, inhibiting 5-lipoxygenase (5-LOX) activity provides cellular resistance against oxidative stress. As a result, 5-LOX is an essential research target. There are numerous antimicrobial processes, such as dihydrofolate reductase, DNA Gyrase B, Penicillin-Binding Protein 2a (PBP2a) enzyme, and Aminoacyl-tRNA synthetases. Dihydrofolate reductase (DHFR) is a well-known molecular target in a variety of therapeutic domains, including cancer treatment and anti-infective medication development. It is essential in the synthesis of antibacterial, antifungal, and antiparasitic compounds. DNA gyrase B is a topoisomerase that is found in almost all bacteria. Its role in DNA replication, repair, and decatenation is critical. Inhibiting penicillin-binding protein 2a (PBP2a) is a promising approach to eliminating methicillin resistance in Staphylococcus aureus (MRSA). Aminoacyl-tRNA synthetases are now recognized as promising molecular targets for antibiotic development. Aminoacyl-tRNA synthetases catalyze the attachment of amino acids to their respective transfer RNAs, which is essential in the protein synthesis process. These enzymes are involved in the first phases of genetic code translation.

The results obtained thus far encourage us to examine the antioxidant activity of the separated flavonol glycosides (metabolites 1–4) derived from the plant under study. This assessment aims to ascertain their potential as agents against MRSA, bolstered by a comprehensive in silico analysis.

## Materials and methods

### Plant material

The fresh leaves and flowers of *Crotalaria madurensis* R. Wight were collected from Al-Azhar Park, Salah Salem St, El-Darb El-Ahmar, Cairo Governorate in May 2019. Egypt (30.0408° N, and 31.2647° E). The identification, authentication, and voucher specimen of the collected plant were established according to the reported (Hala et al. 2008). On July 14th, 2010, the plant name was verified at http://www.theplantlist.org/.

### Extraction and isolation

An amount of 500 g of powdered air-dried aerial part (leaves and flowers) was separately extracted with 70% aqueous methanol (3 × 1 L, 70 °C); each extract was dried via a rotatory evaporator (Buchi Co., Switzerland) to afford 125 g of total extract (25%). Then defatted using hot petroleum ether under reflux (2 × 1L, 60 °C), then taken with ethyl acetate (3 × 1L, 60^◦^C) to extract the flavonoids. After the removal of ethyl acetate on a rotatory evaporator. Thereafter, the ethyl acetate-soluble portion of aerial extract (100 g) was applied to a polyamide (S6) column chromatography (CC) using H_2_O, followed by H_2_O/MeOH step-gradient up to pure MeOH. Using PC, UV light, and spray reagents, similar fractions (I-III) are collected. Fraction (I) was isolated on a cellulose column and eluted with 20–40% aqueous EtOH, then fractionated on a Sephadex column using EtOH to obtain metabolites 1 and 2. Fraction (II) was subjected to Sephadex LH-20 column and eluted via EtOH to produce metabolite 3. Fraction (III) was purified via Sephadex LH-20, cellulose columns with several suitable solvent systems yielding metabolite 4. ^1^H and ^13^C NMR spectral data were performed in the NMR unit at the Faculty of Pharmacy Cairo University in Egypt by using Bruker High-Performance Digital FT-NMR Spectrometer Advance III (400 MHz for ^1^H &100 MHz for ^13^C) (Founded 1960 in Germany). ESI–MS negative ion acquisition mode was carried out on an XEVO TQD triple quadruple instrument (Waters Corporation, Milford, MA01757 U.S.A, mass spectrometer).

### Chromatographic properties of isolated metabolites

Metabolites 1 (50 mg) and 2 (35 mg), yellow amorphous powders, have R_f_ values of 0.50 and 0.20 (15% acetic acid; AA) on PC, respectively, and change color under UV light and with NH3 vapors and AlCl_3_. Metabolite 3, a 20 mg yellowish-white powder, has an R_f_ value of 0.24 (15% AA) and similar chromatographic properties to compound 2. Metabolite 4, a 25 mg yellowish powder, has an Rf value of 0.45 (15% AA) on PC, and changes color under UV light, with FeCl_3_, and with ammonia vapors or alcoholic aluminum chloride solution. NMR data are in Table 1.

### Assessing antioxidant activity in-vitro using various methods

Indeed, Total Antioxidant Capacity (TAC) is evaluated using a variety of assays, including the Ferric Reducing Antioxidant Power (FRAP) and 2,2′-Azino-bis (3-ethylbenzothiazoline-6-sulfonic acid) (ABTS) assays (Lahmass et al. 2018). Detailed methodologies for determining the reducing power capability, FRAP, and ABTS radical scavenging assays have been reported in previous studies (Ahmed et al. 2023; Marzouk et al. 2023). These assays provide valuable insights into the antioxidant potential of various samples.

### The antibacterial activity

#### The antibiotics susceptibility test of methicillin-resistant *S. aureus* ATCC 6538 (MRSA)

The Bauer-Kirby disc diffusion technique was used in conjunction with the National Committee for Clinical Laboratory Standards' techniques (Kiehlbauch et al. 2000) to evaluate the antibacterial susceptibility or resistance of MRSA to various antibiotics. Sigma Aldrich supplied all the antibiotic discs. The antibiotic disc diameters and doses were following World Health Organization (WHO) guidelines. On the nutrient agar plate surfaces, the five antibiotic discs Levofloxacin LEV-5, Cefuroxime CXM-30, Rifaximin RF-30, Streptomycin S-10, and ampicillin/sulbactam SAM-20 (g or unit/disc) were inserted. The agar plates were incubated at 37 °C for 24 h. The inhibition zone around the discs was measured in millimeters (mm).

#### Evaluating the antibacterial impact of plant extract and metabolite 1 on MRSA using the well agar diffusion method

The Agar Well Diffusion technique, a common method for assessing antimicrobial activity, was used to evaluate the antibacterial properties of *C. madurensis* plant extract and metabolite 1 against methicillin-resistant *S. aureus* (MRSA) ATCC 6538. This followed the procedure detailed by Salem et al., 2021. The MRSA strain was kindly supplied by the Drug Microbiology Lab at the National Center for Radiation Research and Technology (NCRRT), a division of the Egyptian Atomic Energy Authority. Initially, the MRSA was incubated overnight in a Nutrient Broth (NB) medium to promote bacterial growth. Following a 24 h incubation period, the MRSA was further incubated for 2 h to reach the lag phase. The MRSA suspension was then adjusted to a standard 0.5 McFarland concentration, equivalent to an inoculum size of 1.0 × 10^8^ CFU/ml. The surface of the nutrient agar plates was inoculated with 100 μl of the bacterial inoculum. A volume of 1.0 ml of the *C. madurensis* plant extract and metabolite 1, at concentrations of 0.25, 0.5, and 1.0 mg/ml, was introduced into the wells (6 mm in diameter) that were punched on the agar surface using a sterile cork borer. The agar plates were subsequently incubated at 37 °C for 24 h. All experiments were conducted in triplicate. The diameter of the inhibition zone (ZOI) was measured in millimeters (mm) and reported as mean values.

#### Minimum inhibition concentration

Minimum Inhibitory Concentration (MIC) of the *C. madurensis* plant extract and metabolite 1 against MRSA were determined by the two-fold dilution method (Fathy et al. 2020). MRSA inoculum was diluted in NB medium to prepare the bacterial cells concentration of 10^8^ CFU/ml. The plant extract and metabolite 1 concentrations were provided as 1.0, 0.5, 0.25, 0.125, and 0.062 mg/ml. A volume of 100 μl of MRSA suspensions (10^8^ CFU/ml) was introduced to the tubes with treatment dilutions. After a 24 h incubation period at 37 °C, the turbidity of the bacterial growth was observed. The MIC was recognized as the lowest treatment concentration that could inhibit the noticeable MRSA growth after 24 h of incubation.

#### Impact of metabolite 1 and plant extract on MRSA cell survival

The antibacterial activity of the *C. madurensis* plant extract and metabolite 1 on the viability of MRSA was evaluated by Abd-Allah W, Fathy RM.,2022 (Abd-Allah and Fathy 2022). The bacterial suspension was adjusted to a 0.5 McFarland standard. Stock solutions of the plant extract and metabolite 1 were prepared at concentrations of 1.0, 0.5, and 0.25 mg/ml. MRSA without any treatment served as the control sample. Each stock solution underwent a tenfold serial dilution to create dilutions ranging from 10^–1^ to 10^–5^. The tubes were incubated at 37 ºC for 18 h. Subsequently, 100 µl of the three most recent dilutions were spread on nutrient agar plates and incubated at 37 ºC for 24 h. This experiment was repeated three times. Colonies were counted for each dilution, and the CFU/ml was determined. The reduction in viable cells was expressed as log_10_ CFU/ml.

#### Impact of gamma irradiation on the antibacterial efficacy of metabolite 1 and plant extract against MRSA

Test samples of the plant extract and metabolite 1, at concentrations of 1.0, 0.5, and 0.25 mg/ml, were prepared. These samples were divided into two equal parts, sealed in tubes, and individually exposed to 50 and 100 Gy of radiation using the Indian Gamma Cell at the National Centre for Radiation Research and Technology (NCRRT), part of the Egyptian Atomic Energy Authority in Cairo, Egypt. This was done at a dose rate of 0.704 kGy/h. The antibacterial activity of the irradiated plant extract and metabolite 1 against MRSA was then assessed using the well-agar diffusion method.

#### Analyzing the differences in Fourier-transform infrared spectra between the control and treated MRSA filtrate

Fourier-Transform Infrared (FTIR) analysis was conducted on the filtrates of both untreated MRSA and MRSA treated with plant extract and metabolite 1. This was done to identify changes in wave number and transmittance percentage, which could indicate the treatments’ binding to the bacterial cells. Prior to the analysis, the control and bacterial pellets were thoroughly grounded with Potassium Bromide (KBr) to achieve a 2% concentration. The analysis covered the entire spectral region from 400 to 4000 cm^−1^. The background, corresponding to pure KBr, was subtracted from each sample’s spectra.

### Statistical analysis

All results were analyzed using one-way ANOVA at p < 0.05. The columns represent the mean values of the results, and the error bars represent standard deviations (SD). The data were examined by IBMCorp (Released 2016. IBM SPSS Statistics for Windows, Version 24.0. Armonk, NY: IBM Corp). References and citations were compiled using the EndNote software.

### Molecular docking

The docking analysis was performed using the Autodock Vina software. Enzymes like lipoxygenase, dihydrofolate reductase, DNA gyrase B, penicillin-binding protein 2A, and threonyl-tRNA synthetase were sourced from the Protein Data Bank (PDB). The ligands, protein, and docking parameters files were created using the reported procedure (Harras et al. 2023; Soleman et al. 2023). To visualize and analyze the protein–ligand interactions within the active site of the complex, the Biovia Discovery Studio 2021 visualizer was utilized.

## Results

### Phytochemistry

**Fig. 1:**
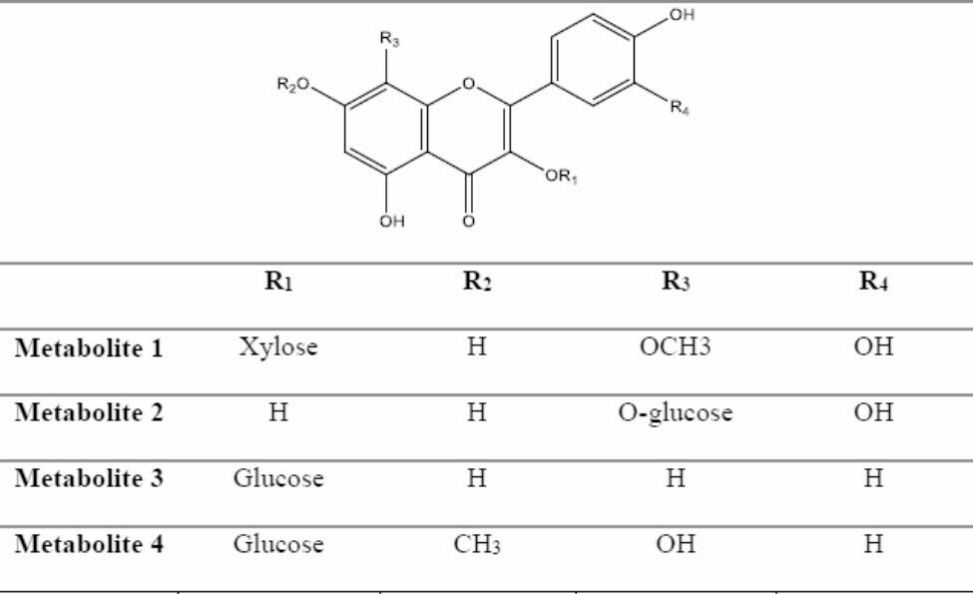
The chemical structure of isolated metabolites from *C. madurensis*

Four compounds were extracted from the alcoholic extract of *C. madurensis* through a variety of chromatographic methods. The identification of these compounds was accomplished using spectral analysis techniques like ^1^H NMR, ^13^C NMR, and ESI–MS (refer to Fig. 1 and Table 1).

**Table 1:** ^1^H and ^13^CNMR spectral data of isolated metabolites (400/100 MHz, DMSO-*d*_*6*_)*

No.	Metabolite1		Metabolite 2		Metabolite 3		Metabolite 4	
	^1^H-NMR δ_HJ (Hz)_	^13^C-NMR δ_C_	^1^H-NMR δ_HJ (Hz)_	^13^C-NMR δ_C_	^1^H-NMR δ_HJ (Hz)_	^13^C-NMR δ_C_	^1^H-NMR δ_HJ (Hz)_	^13^C-NMR δ_C_
2	–	156.76	–	156.04	–	155.99	–	155.97
3	–	132.99	–	132.98	–	132.88	–	132.94
4	–	177.98	–	177.81	–	177.71	–	177.72
5	–	156.05	–	156.84	–	162.78	–	162.77
6	6.25 (s)	99.19	6.11 (s)	99.11	6.44 (d, *J* = 2.2 Hz)	98.10	6.35 (s)	98.17
7	–	156.05	–	156.84	–	163.75	–	165.78
8	–	126.13	–	126.20	6.20 (d, *J* = 2.2 Hz)	93.73	–	126.15
9	–	149.33	–	149.26		156.76	–	156.76
10	–	103.79	–	102.40		104.65	–	104.65
1`	–	121.55	–	121.59		121.50	–	121.96
2`	7.88 (brs)	116.34	7.89 (brs)	117.42	7.87 (d, *J* = 8.4 Hz)	130.72	7.87 (d, *J* = 8.3 Hz)	130.72
3`	–	145.62	–	145.57	6.88 (d, *J* = 8.3 Hz)	115.70	6.80 (d, *J* = 8.4 Hz)	115.73
4`	–	148.87	–	148.72	-	160.118	-	160.13
5`	6.85 (d, *J* = 7.6 Hz)	115.16	6.84 (d, *J* = 8.4 Hz)	115.26	6.88 (d, *J* = 8.3 Hz)	115.70	6.80 (d, *J* = 8.4 Hz)	115.73
6`	7.82 (d, *J* = 7.6 Hz)	121.56	7.83 (d, *J* = 8.4 Hz)	121.79	7.87 (d, *J* = 8.4 Hz)	130.72	7.87 (d, *J* = 8.3 Hz)	130.72
1``	5.71 (d, *J* = 6.8 Hz)	102.36	5.75 (d, *J* = 7.6 Hz)	104.81	5.18 (d, *J* = 7.7 Hz)	102.28	5.18 (d, *J* = 8.6 Hz)	102.27
2``	3.17–3.37 (m) Remaining of sugar protons	73.74	3.13–3.38 (m) Remaining of sugar protons	73.79	3.12–3.39 (m) Remaining of sugar protons	73.30	3.18–3.36 (m) Remaining of sugar protons	73.31
3``		76.60		76.99		76.66		76.82
4``		69.75		70.45		70.26		69.04
5``		66.03		77.39		77.28		77.27
6``		–		61.24		61.03		61.06
7-OCH_3_	–	–		–		–	3.78 (s)	56.10
8-OCH_3_	3.74(s)	56.20		–		–		–

* *J*;:coupling constant, s: singlet, d: doublet, brs: broad singlet, m: multiplet.

### Antioxidant screening

**Fig. 2:**
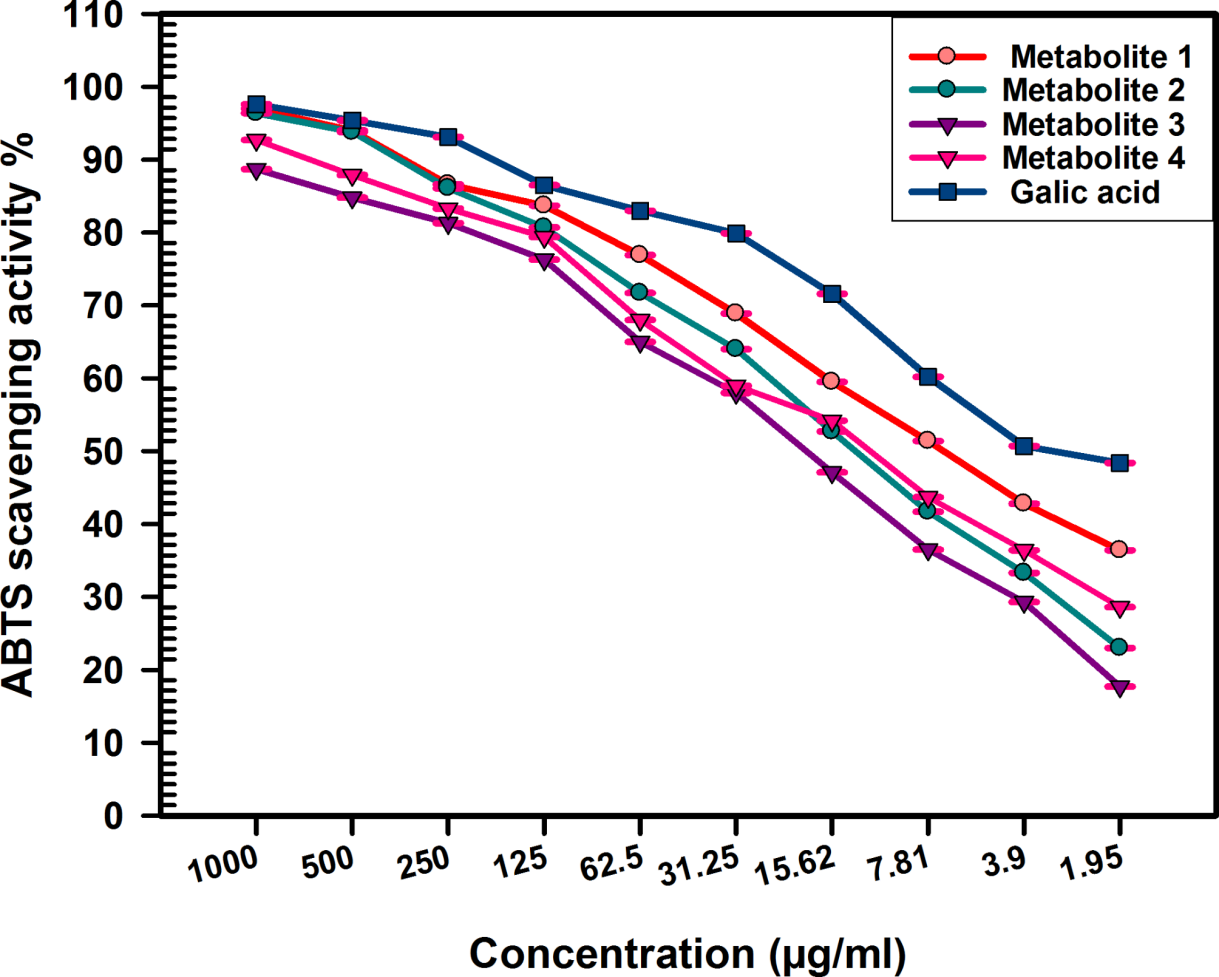
ABTS radical scavenging activity of *C. madurensis* metabolites1-4 compared to gallic acid as a reference drug. Data were analyzed using one-way analysis of variance (ANOVA) and were expressed as Mean ± SD (n = 3)

**Fig. 3:**
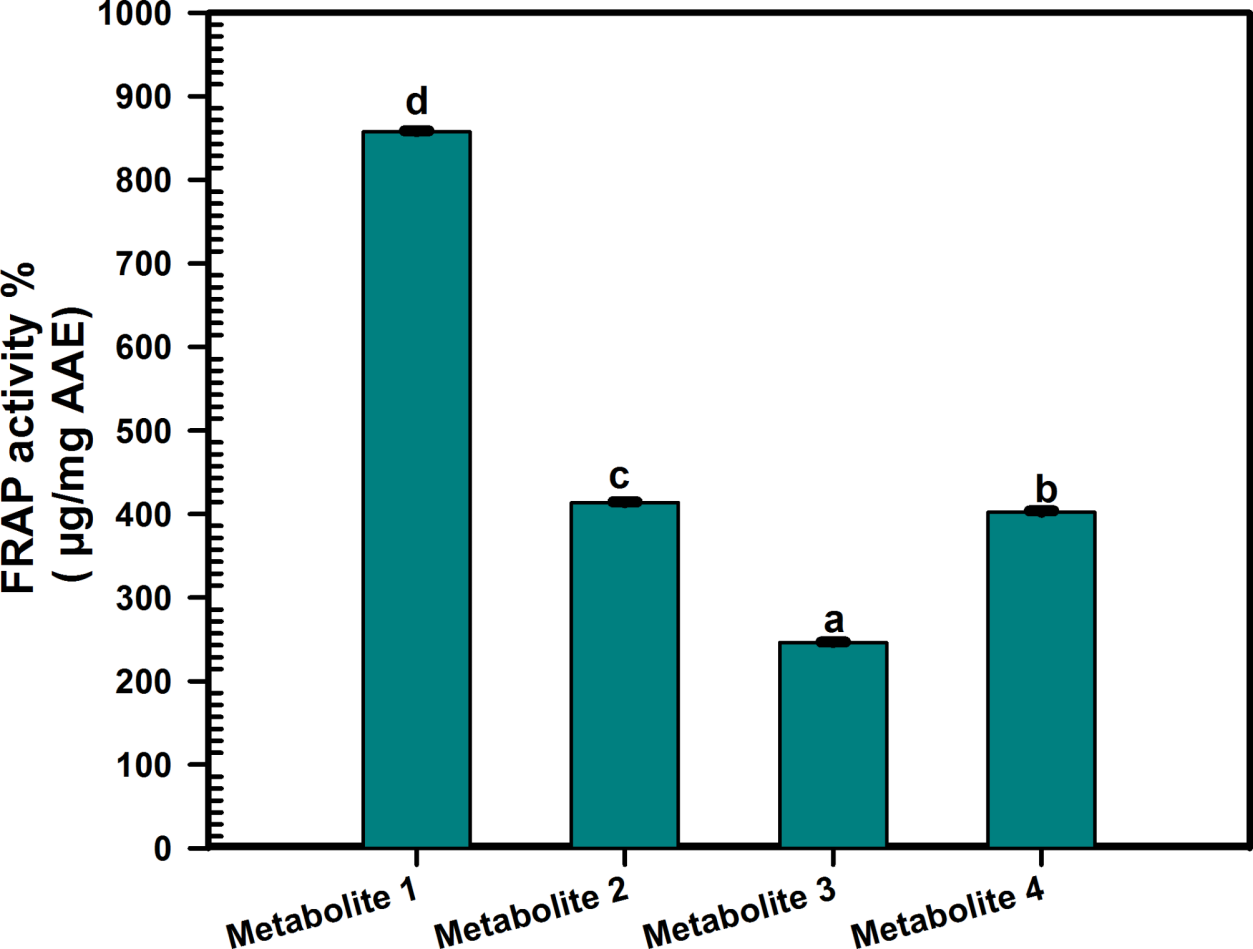
Ferric reducing power ability of *C. madurensis* metabolites (1–4). Data were analyzed using one-way analysis of variance (ANOVA) and expressed as Mean ± SD (n = 3). Means bearing different letters differ significantly at p ≤ 0.05 by Duncan’s multiple range test

**Fig. 4:**
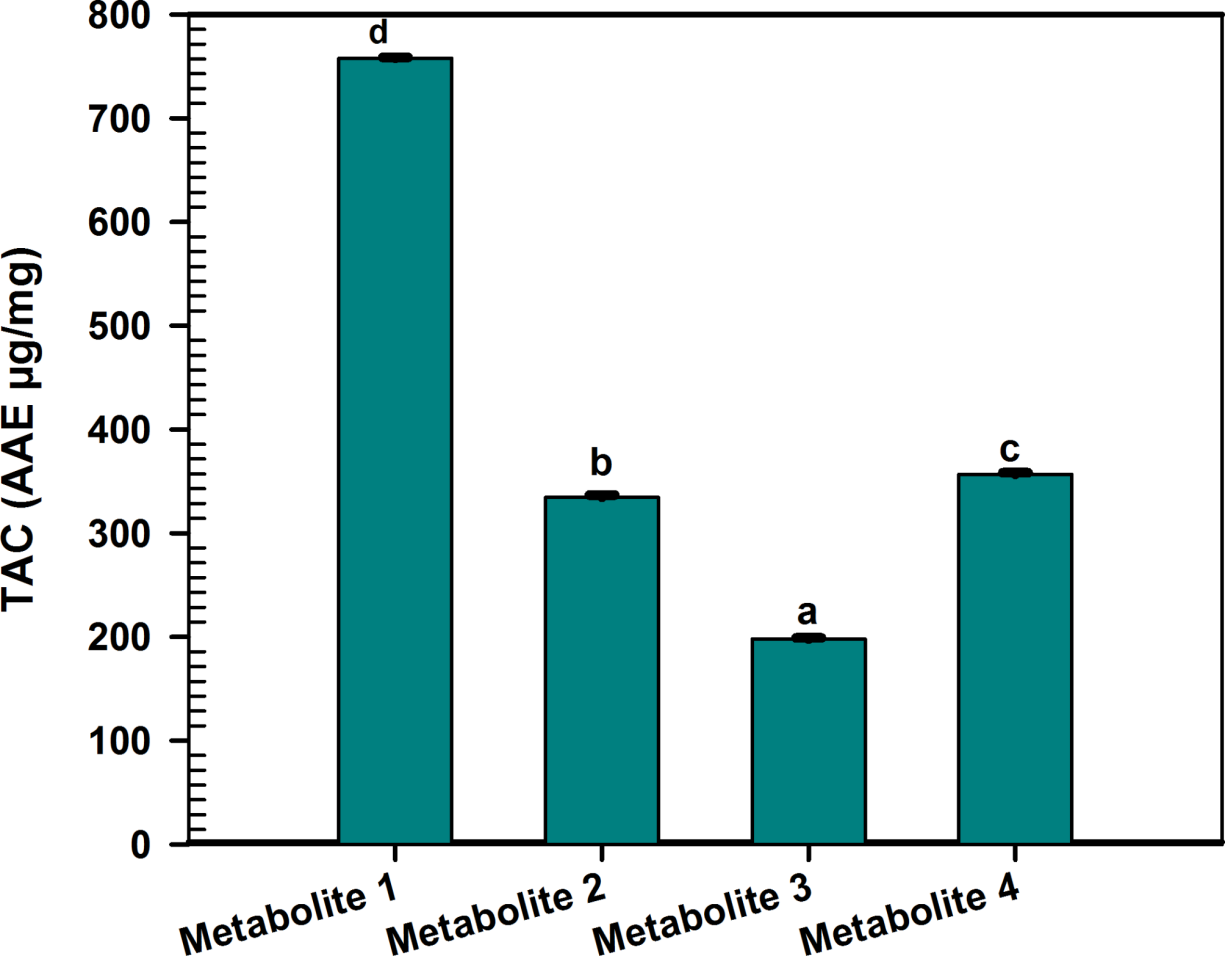
Total antioxidant capacity assay of *C. madurensis* metabolites (1–4). Data were analyzed using one-way analysis of variance (ANOVA) and expressed as Mean ± SD (n = 3). Means bearing different letters differ significantly at p ≤ 0.05 by Duncan’s multiple range test

Prior studies have validated the antioxidant activity of the *C. madurensis* extract (Hala et al. 2008). This research focuses on the antioxidant capabilities of metabolites derived from the aerial parts of the plant. The antioxidant impact of these metabolites was evaluated using a range of assays, as depicted in Figs. 2, 3, 4.

### Scavenging activity of ABTS radical cation (RSA%)

The ABTS assay is based on the conversion of the ABTS radical into a non-radical form through oxidation with potassium persulphate. This process changes color when samples with antioxidant properties are added.

In this context, metabolite 1 demonstrated the highest free radical scavenging activity, as evidenced by the ABTS scavenging activity percentage and the lowest IC_50_ value of 6.993 µg/mL (Fig. 2). This aligns with the antioxidant activity observed in all the samples examined. Figure 2 illustrates those metabolites 4, 2, and 3 also have significant antioxidant potential, with IC_50_ values of 12.23, 13.90, and 17.92 µg/ml, respectively. For comparison, the IC_50_ value of the standard gallic acid was found to be 3.460 µg/ml. Notably, metabolite 1 surpassed the others in scavenging activity across all concentrations (1.95–1000 µg/ml), underscoring its superior antioxidant potential.

### Ferric reducing antioxidant power (FRAP) assay

This study assessed the iron-reducing activity of samples based on an antioxidant’s ability to transfer electrons from Fe^3+^ to Fe^2+^. A higher absorbance value signifies a greater antioxidant capacity of tested samples. The results showed an increase in the FRAP of metabolites 1–4, indicating their reducing potential. This analysis is based on the increase in the reaction mixture’s absorbance (OD at 630 nm). All metabolites demonstrated some degree of power reduction (Fig. 3), with metabolite 1 showing the highest FRAP ability (857.7 ± 1.015 µg/mg). Metabolites 2 and 4 exhibited moderate ferric-reducing power activity (413.6 ± 1.002, 402.2 ± 1.457 µg/mg, respectively), while metabolite 3 recorded the lowest activity (246.2 ± 0.6506 µg/mg).

### Total antioxidant capacity assay (TAC)

In terms of Total Antioxidant Capacity (TAC), the results mirrored the pattern observed in the FRAP activity (Fig. 4). Metabolite 1 was distinguished by its superior antioxidant capacity, registering a value of 757.8 ± 0.7937 µg/mg. Both Metabolites 4 and 2 also showcased notable antioxidant activity, with respective values of 356.8 ± 1.480 µg/mg and 334.6 ± 2.179 µg/mg. Conversely, Metabolite 3 had the least antioxidant capacity, recording a value of 197.8 ± 1.179 µg/mg. These observations underscore the diverse antioxidant capacities of the metabolites, with Metabolite 1 emerging as the most potent.

### The antibacterial activity

#### The susceptibility test for antibiotics on the methicillin-resistant strain of *S. aureus* (MRSA)

**Fig. 5:**
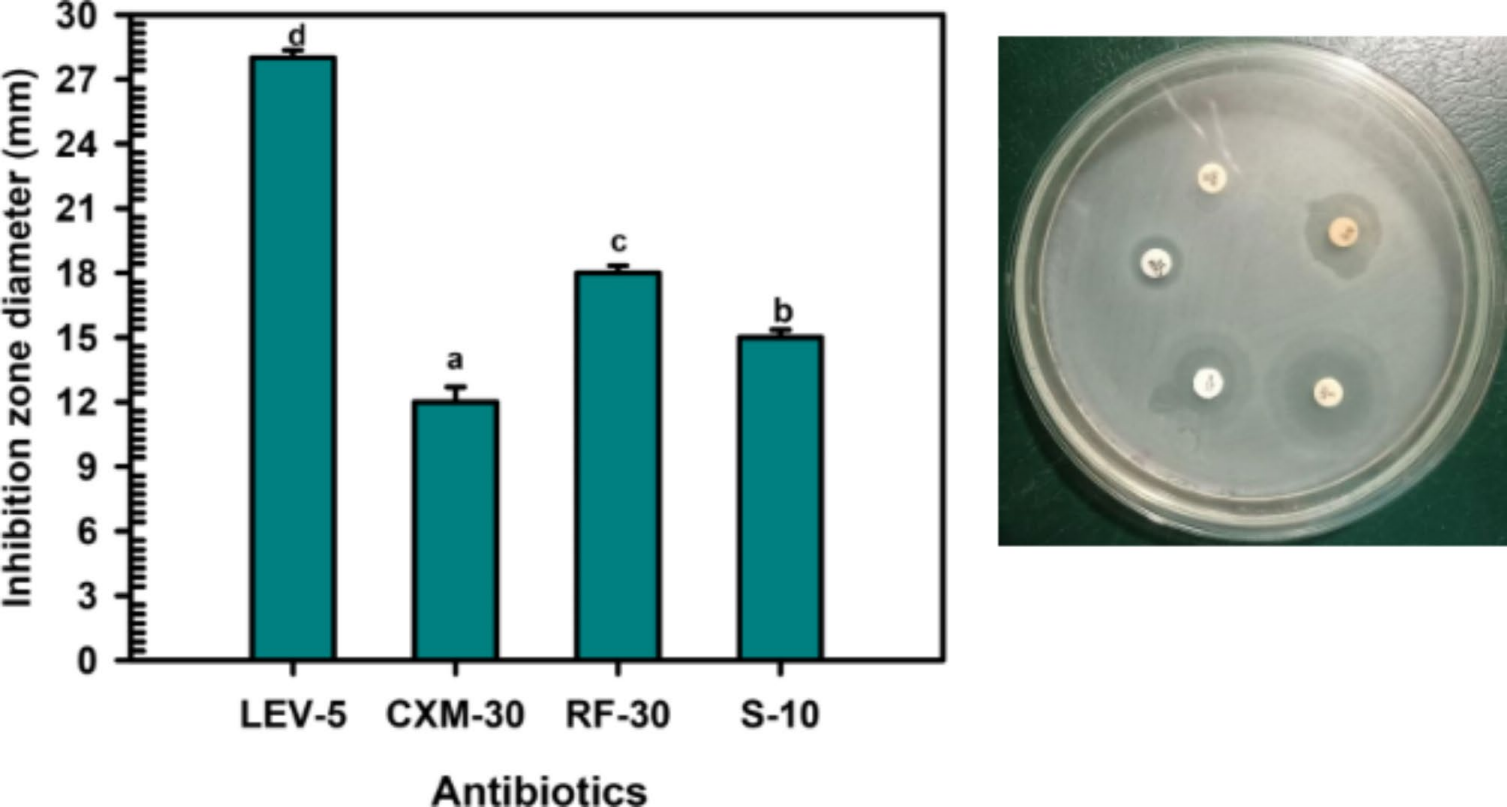
The response of MRSA to antibiotics like levofloxacin, cefuroxime, rifaximin, streptomycin, and ampicillin/sulbactam SAM-20

An antibiotic susceptibility test was performed on methicillin-resistant *S. aureus* (MRSA) using five different antibiotics. The results showed that most of these antibiotics were effective in inhibiting MRSA growth. These antibiotics, which belong to various groups and work through different mechanisms, were found to be either susceptible or resistant to MRSA. The inhibition zones’ diameters were ranked in descending order as follows: Levofloxacin LEV-5 (29.41 ± 1.03 mm), Rifaximin RF-30 (18.0 ± 0.65 mm), Streptomycin S-10 (15.27 ± 0.54 mm), and Cefuroxime CXM-30 (12.36 ± 0.82 mm). However, MRSA showed resistance to Ampicillin/Sulbactam SAM-20 (as shown in Fig. 5).

#### Assessing the antibacterial impact of plant extract and metabolite 1 on MRSA via the well agar diffusion method

Antimicrobial agents are commonly used to treat microbial infections caused by medical conditions, environmental factors, and/or food poisoning, with MRSA infection being a key example. Recently, there has been a significant interest in plant flavonoids due to their potential to fight pathogenic microbes (Okwu et al. 2019). This study examines the antibacterial effects of the *C. madurensis* extract and its metabolite 1 using the well agar diffusion technique. The plant extract and its metabolite 1 were tested for MRSA at concentrations of 1.0, 0.5, and 0.25 mg/ml.

**Fig. 6:**
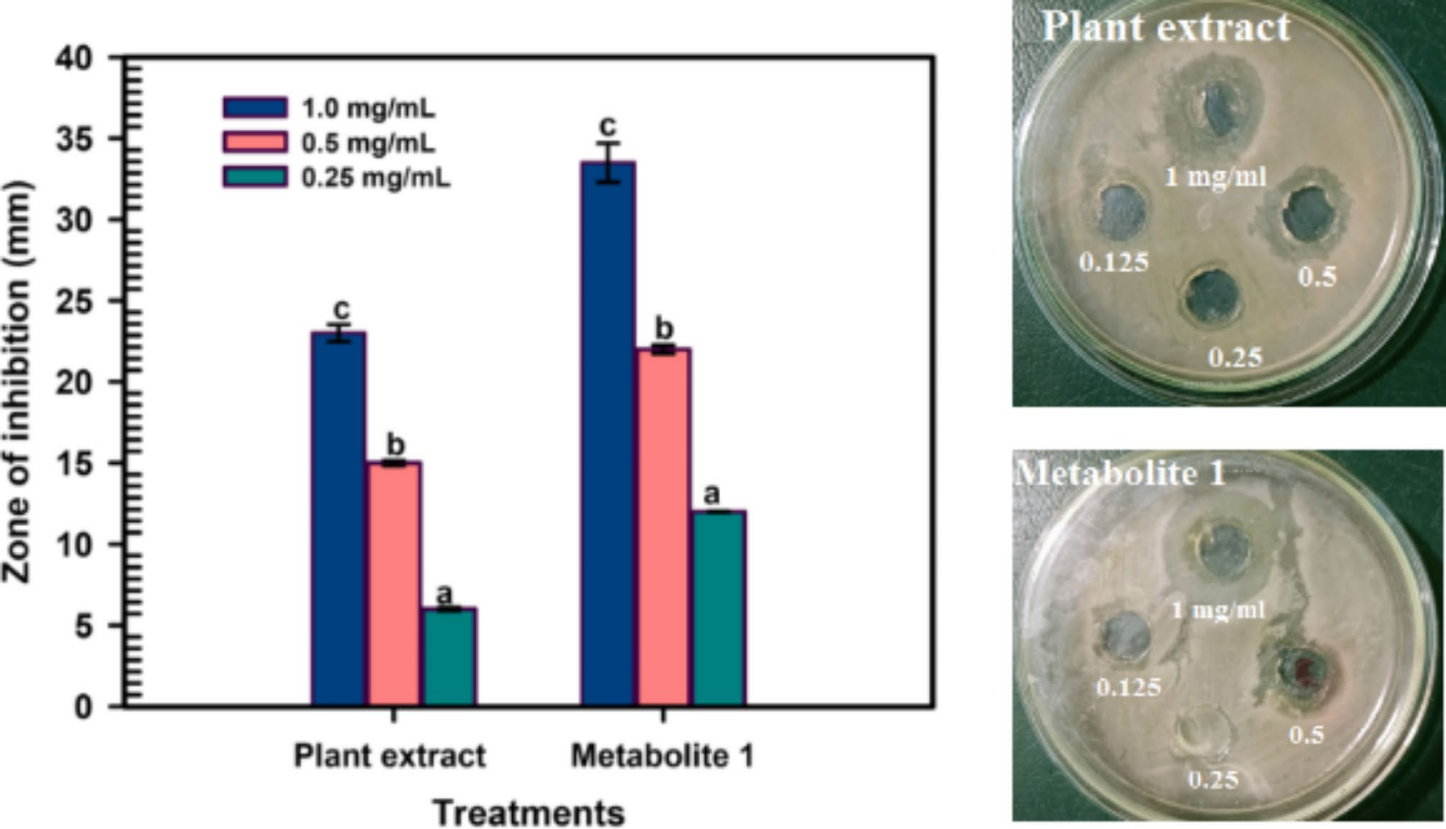
The antibacterial effect of the plant extract and metabolite 1 on MRSA at concentrations of 1.0, 0.5, and 0.25 mg/ml, with the error bar indicating the standard error (n = 3). Means bearing different letters differ significantly at p ≤ 0.05 by Duncan’s multiple range test

As shown in Fig. 6, the plant extract showed substantial antibacterial activity at concentrations of 1.0 and 0.5 mg/ml, with inhibition zone diameters of 23 ± 0.521 and 15 ± 0.127 mm, respectively. Metabolite 1 had the most potent antibacterial effect on MRSA, with inhibition zone diameters of 33.5 ± 1.20, 22.59 ± 0.261, and 12.00 ± 0.036 mm at concentrations of 1.0, 0.5, and 0.25 mg/ml, respectively.

The Minimum Inhibitory Concentration (MIC) dilutions ranging from 0.062 to 1.0 mg/ml of the *C. madurensis* extract and metabolite 1 were assessed against MRSA. The MIC for the plant extract was found to be 0.5 mg/ml, while for metabolite 1, it was 0.25 mg/ml.

#### The impact of plant extract and metabolite 1 on the survival of MRSA cells

**Fig. 7:**
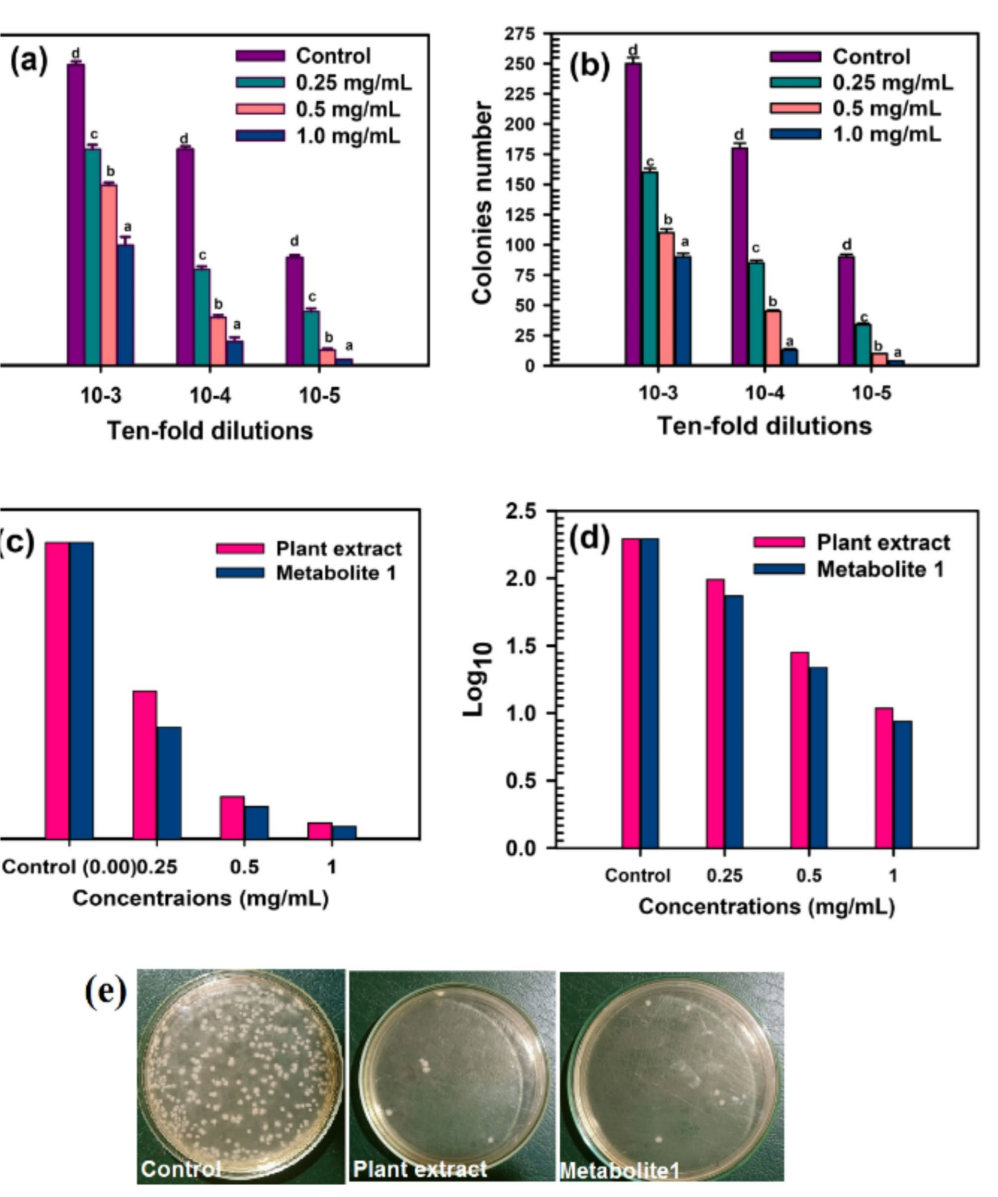
The MRSA viability is depicted as follows: **a** The number of colonies after treatment with plant extract. **b** The number of colonies after treatment with metabolite 1. **c** The count of Colony Forming Units (CFU) per milliliter, and **d** The base-10 logarithm. **e** The count of colonies from the 10^–5^ dilution at a concentration of 1.0 mg/ml on Nutrient Broth (NB) medium. Means bearing different letters differ significantly at p ≤ 0.05 by Duncan’s multiple range test

A viable cell count test was conducted to evaluate the impact of the plant extract and metabolite 1 at concentrations of 1.0, 0.5, and 0.25 mg/ml on the survival of MRSA cells following overnight exposure. The results were expressed as colony numbers, colony-forming units per millimeter (CFU/ml), and log_10_. The data revealed the effective inhibitory activity of the plant extract and metabolite 1 on MRSA viability. Metabolite 1 exhibited superior antibacterial activity against MRSA across all used concentrations, surpassing the activity of the plant extract. Bacterial inhibition was estimated within the range of 10^–4^ to 10^–5^ ten-fold serial dilutions of the MRSA inoculum. As depicted in Fig. 7, the decrease in colony-forming units begins after the 10^–3^ dilutions and progressively increases as the concentration rises. With metabolite 1, complete inhibition occurs at 10–5 dilutions for 1.0 mg/ml, while a significant reduction was observed with the plant extract.

As expected, CFU/ml values heavily depend on the colony number results, so the CFU/ml inversely correlates with the treatment concentrations, decreasing as the concentrations increase. Furthermore, metabolite 1 at a concentration of 1.0 mg/ml records the lowest CFU/ml (0.04 × 10^8^), compared to the plant extract (0.05 × 10^8^) and the untreated control (0.9 × 10^8^) at the same concentration (Fig. 7).

The log_10_ values in Fig. 7 corroborate the results obtained from the cell count, where 1.0 mg/ml of plant extract and metabolite 1 record log_10_ of 4.18 and 3.25, respectively, compared to the log_10_ of the untreated control (7.451).

#### Impact of gamma irradiation on the antibacterial efficacy of the plant extract and the extracted flavonoid against MRSA

**Fig. 8:**
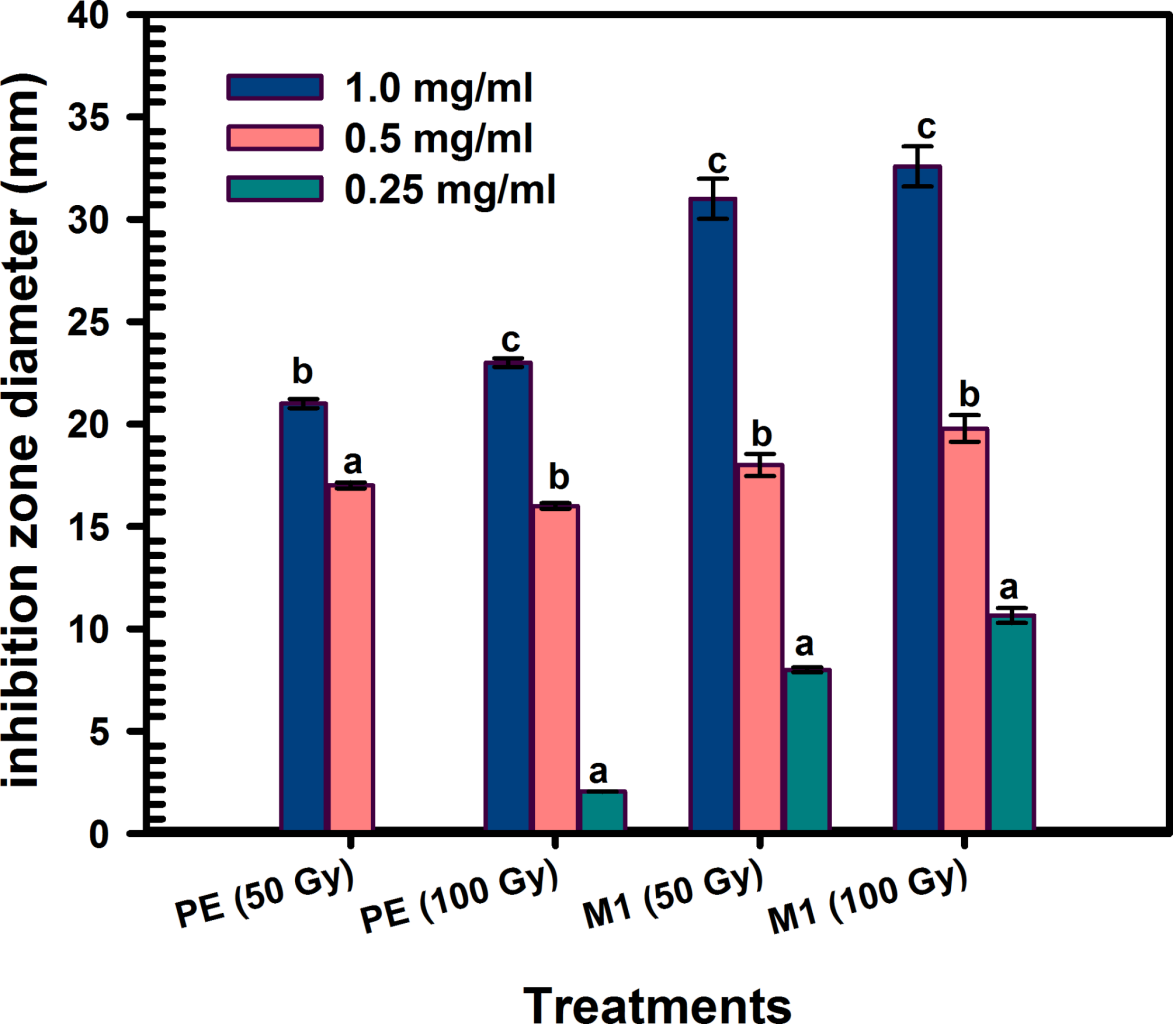
Impact of gamma irradiation at doses of 50 and 100 Gy on the antibacterial efficacy of plant extract (PE) and metabolite 1 (M1) against MRSA

The impact of gamma irradiation on the antibacterial activity of the *C. madurensis* plant extract (PE) and metabolite 1 (M1) against MRSA was evaluated. The flavonoid samples were subjected to irradiation at 50 and 100 Gy, as depicted in Fig. 8. The results indicate that gamma irradiation does not enhance the inhibitory effect of the plant extract and metabolite 1 on MRSA compared to the non-irradiated samples. The most pronounced effect of gamma irradiation is observed at 100 Gy and 1.0 mg/ml for the plant extract and metabolite 1, respectively, with inhibition zone diameters of 23.0 ± 0.214 and 32.58 ± 0.95, respectively.

#### Contrasting the Fourier-transform infrared spectra of the untreated and treated MRSA filtrate

**Fig. 9:**
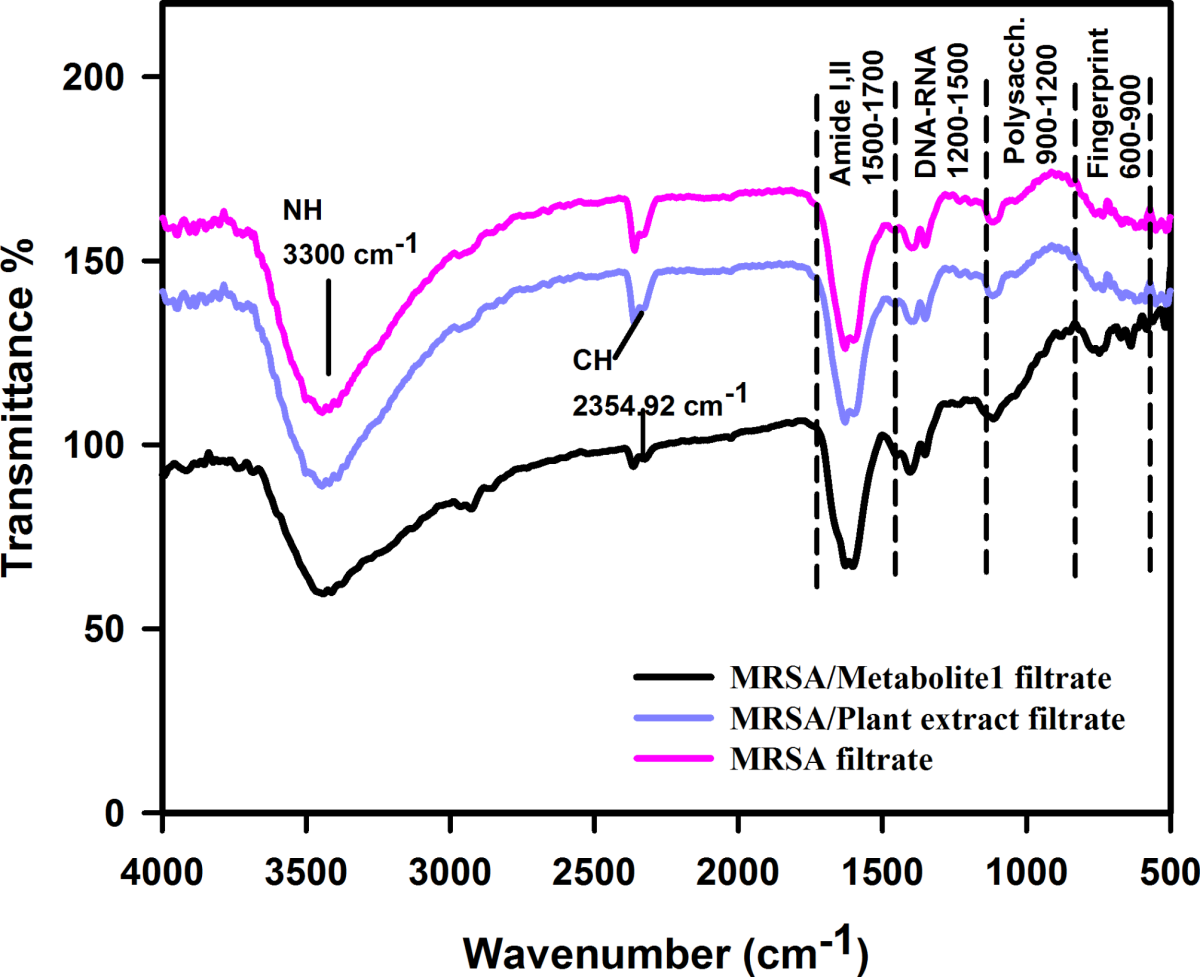
FTIR of the filtrates from MRSA, MRSA treated with plant extract, and MRSA treated with metabolite 1

The FTIR study was conducted to illustrate the spectral changes in MRSA filtrates that were untreated (serving as a control) and those treated with plant extract and metabolite 1. The FTIR spectral data was gathered in the frequency band of 400–4000 cm^−1^. As depicted in Fig. 9, there are significant differences in band frequency values, bandwidth, and peak intensity (%) between the control and treated spectra. The application of plant extract and metabolite 1 notably decreases the transmittance (%) across all vibration bands, suggesting a reduction in vital bacterial cell components such as protein, polysaccharides, and phospholipids content. As demonstrated in Fig. 9, the intensity of the stretching vibration was higher in the untreated filtrate compared to the treated ones.

#### Molecular docking

The objective of docking is to precisely predict a ligand's conformation within a binding site and evaluate its binding affinity. Docking simulations were employed to assess the affinity between four metabolites (gossypetin 8-methoxy, 3-*O*-β-D-xylopyranoside, gossypetin 8-*O*-β-D-glucopyranoside, kaempferol 3-*O*-ß-D-glucoside (Astragalin), and herbacetin7-methyl ether-3-*O*-β-D-glucopyranoside) and the binding sites of arachidonate-5-lipoxygenase (PDB: 6n2w) (Ley-Martínez et al. 2022). This study aimed to reveal the potential antioxidant mechanism of these metabolites. Docking scores indicated that all four metabolites had lower binding energy values compared to the co-crystal ligand (Table S1, supplementary data) of these, gossypetin 8-methoxy, 3-*O*-β-D-xylopyranoside (metabolite 1) had the highest score.

The antimicrobial effectiveness of this metabolite implies that its mechanism of action might involve interactions with key enzymes that aid in bacterial eradication, such as dihydrofolate reductase (PDB ID: 3sr5) (Li et al. 2011), DNA gyrase (PDB ID: 3g75)(Ronkin et al. 2010), penicillin-binding protein (PBP2a) (PDB ID: 1mwt) (Lim and Strynadka 2002), and threonyl-tRNA Synthetase (PDB ID: 1nyq) (Torres-Larios et al. 2003). These findings are detailed in (Table S2, supplementary data).

The co-crystallized ligands were re-docked into their respective enzymes, resulting in a root-mean-square deviation (RMSD) of 0.62 Å (Lipoxygenase enzyme), 0.12 Å (dihydrofolate reductase), 0.08 Å (DNA gyrase), 0.70 Å (PBP2a), and 0.65 Å (threonyl-tRNA synthetase) between the docked and co-crystallized ligand (Figures S15–S19, supplementary data). This confirms the validity of the docking methodology used.

## Discussion

*C. madurensis*, renowned for its medicinal secondary metabolites like flavonol glycosides, offers a promising avenue for future research in natural product-based therapeutics(Yaquba et al. 2020). The glycoside form of these metabolites enhances their water solubility, reduces toxicity and side effects, and improves targeting, making them particularly valuable(Khodzhaieva et al. 2021). The synthesis of various flavonol glycosides opens a world of possibilities for exploring their medicinal properties. In this context, four compounds, presumed to be flavonol *O*-glycosides (1–  4), were isolated from the aerial part of *C. madurensis* using sequential column chromatography. These compounds demonstrated consistent chromatographic characteristics, such as fluorescence in short/long UV light, Rf values, and their changes with NH3 vapors, AlCl3, and FeCl3, highlighting the potential of these compounds and emphasizing the need for ongoing research in this field. On the PC chromatogram, metabolites 1, 3, and 4 were identified as deep purple spots that transitioned to yellow fluorescence when exposed to NH3 vapors and AlCl3. These compounds turned green when treated with FeCl3, suggesting they are likely flavonol 3-*O*-glycoside structures. Metabolite 2, on the other hand, was seen as a yellow, fluorescent spot under UV light. It turned yellow when exposed to ammonia vapors and green when sprayed with FeCl3 reagent, indicating it is likely a flavonol glycoside with a free C3-OH group. Furthermore, when treated with the Naturstoff spray reagent, their spots changed to orange (for metabolites 1, 2) and greenish-yellow (for metabolites 3, 4), confirming the aglycones as quercetin or kaempferol, respectively (Markham 1982; Agrawal and Olsson 1989).

Given the chromatographic characteristics, it was inferred that metabolite 1 likely possesses a structure similar to that of 3-*O*-glycosyl quercetin (Markham 1982). NMR studies provided additional structural evidence. The ^1^H NMR spectrum (refer to Table 1) displayed a unique ABM-spin coupling system for H-2′, 6′, and 5′ at δ7.88 (br s), 7.82 (d, *J* = 7.6 Hz) and 6.85 (d, *J* = 7.6 Hz). This is indicative of a 3’,4’-dihydroxy ring B of a quercetin-like aglycone. The absence of H-8 and a singlet signal at δ 6.25, attributable to H-6, suggest that the aglycone is a 3, 5, 7, 8, 3′, 4′ hexahydroxy flavone (gossypetin) (Yaque et al. 2016).

The stereo structure of glycoside moiety was established as β -D-xylopyranoside because of the characteristic δ**-**values and splitting pattern of its anomeric proton signal at 5.71 (d, *J* = 6.8 Hz, H-1′′). Also, the presence of a characteristic signal assigned at δ_H_ 3.74 which is assignable for the methoxy group. Accordingly, the structure of metabolite **1** was established as quercetin 3-*O*-^4^C_1_- β -D-xylopyranosyl methoxyl ether. Final structure confirmation was achieved from the ^13^C NMR spectrum (Table 1) which showed 15 C-resonances of quercetin moiety were interpreted including the key signals at ppm 177.9, 148.8, 145.6, and 132.9 of C-4, C-4′, C-3′, and C-3, respectively. In addition, the xylopyranoside moiety was further established by the key ^13^C-resonances at δ 102.3, 73.7, 76.6, 69.7, and 66.0 for the anomeric carbon, C-2", 3", 4" and 5", respectively(Mizuno et al. 2022). The location of C-3 at 132.9 was a confirmative document for the glycosidation at OH-3. In addition to the downfield shift of C-8 to 126.1 (∆ +  ~ 32 ppm) and upfield shift of C-5, 7, and 9 to 156.0, 156.0, and 149.3, respectively (∆ +  ~ 8 ppm) indicate methoxylation at C-8 which causes upfield effects on ^1^H and ^13^C chemical shifts of *ortho* and *para*-position of the flavonol(Lee et al. 2008). While the hydroxylation at the A-ring has no effect on the ^1^H and ^13^C chemical shift changes at the B-ring, and vice versa (Park et al. 2007). Thus metabolite 1 was established as Gossypetin 8-methoxy, 3-*O*-β-D-^4^C_1_-xylopyranoside.

Metabolite 2 was expected to be a quercetin-type *O*-glycoside structure. Further confirmation for the proposed structure was obtained from the ^1^HNMR spectrum (Table 1), which showed an ABM-spin coupling system at ppm 7.89 (br s), 7.83 (d, *J* = 8.4 Hz), 6.84 (d, *J* = 8.4 Hz), interpretable for H-6', H-2' and H-5' of 3,4-dihydroxy B-ring. The absence of H-8 and a singlet signal at δ 6.25 assignable to H-6 suggest that the aglycone is 3, 5, 7, 8, 3', 4' hexahydroxy flavone (gossypetin) (Yaque et al. 2016). The stereo structure of glycoside moiety was established as ^4^C_1_- β -D-glucopyranoside because of the characteristic δ**-**values and splitting pattern of its anomeric proton signal at 5.75 (d, *J* = 7.6 Hz, H-1′′). Accordingly, the structure of metabolite **2** was established as quercetin-8-*O*-^4^C_1_- β -D-glucopyranosyl. ^13^C NMR spectrum provided the final structural confirmation, as it displayed gossypetin’s characteristic C- resonances, particularly the key ones at δ 177.8, 148.7, 145.5, and 132.9, corresponding to-4, C-4′, C-3′, and C-3, respectively. with upfield chemical shift of C-5 (156.8), 7 (156.8), 9 (149.33, 149.2) as the sugar substitution at C-8 causes upfield effects on ^1^H and ^13^C chemical shifts of *ortho* and *para*-position of flavonol (Lee et al. 2008) and downfield shift of C-8 (126.2). As well, 6 characteristic resonances agreed with an β-^4^C_1_-D-glucopyranosyl structure of the glucoside moiety at 104.8 (C-1"), 77.3 (C-5"), 76.9 (C-3"), 73.7 (C-2"), 70.4 (C-4") and 61.2 (C-6") (Mizuno et al. 2022). Thus metabolite 2 was established as Gossypetin 8-*O* β-D -glucopyranoside. Metabolite 3 exhibited chromatographic characteristics of flavonol structure(Mabry et al. 1970). On complete acid hydrolysis, it gave kaempferol (organic phase), and hexose (aqueous phase) (Co PC with authentic samples and detection by specific spray reagents). In ^1^H NMR Table 1 an A2 X2- spin coupling system of two ortho doublets, each of two protons, was assigned at 7.87 and 6.88 with J-value of 8.4 Hz for H-2' /6' and 8.3 Hz for H-3' /5' of 1,4-disubstituted β- ring. Also, two meta doublets were established for H-8 and H-6 at 6.20 and 6.44 respectively, with J-value of 2.2 Hz, of 5, 7-dihydroxy A-ring. In the aliphatic region, showed a ß-anomeric proton signal of hexose moiety at 5.18 (*J* = 7.7 Hz) (Wang et al. 2015). ^13^C NMR, Table 1 showed 15 carbon resonances characteristic of kaempferol aglycone among which five key signals at 177.71 (C-4), of C- ring, 160.118 (C-4 ′), 130.72 (C2' /6′), 115.70 (C-3' /5′) of the B-ring and 132.88 (C-3). Also, the presence of six carbon resonances of glucose moiety. Finally, all C-signals of the aglycone and sugar moieties were confirmed by the comparison with those of the structurally related compounds published before (Kazuma et al. 2000). The structure was confirmed by a negative ESI/ MS spectrum that showed m/z 447.0 [M-H]^−^. Therefore, metabolite 3 was identified as Kaempferol 3-*O-ß–D*-glucoside (Astragalin). This is the first time to be isolated from this plant. Metabolite 4 displayed chromatographic properties indicative of a kaempferol structure. The ^1^H NMR data for metabolite 4 revealed three aromatic proton signals at δ_H_ 7.87 (H-2′,6′), 6.80 (H-3′,5′), and 6.35 (H-6). The anomeric proton signal of glucose was observed at δ_H_ 5.18 (*J* = 8.6 Hz), suggesting it is in the β-form due to the coupling constants of *J* = 8.6 Hz. In the ^1^H NMR data of metabolite 3, two distinct patterns of proton resonances were observed. The first pattern was characteristic of kaempferol, with a broad signal at δ_H_ 12.77 ppm for the exchangeable proton of 5-OH with the 4-keto group. According to Table 1, only one carbon resonance was found between δ_C_ 90 and 105 at 98.17 ppm. Additionally, carbon resonances at δ_C_ 126.15 could only be explained by hydroxylation at position 8. The most up-field peak was present at δ_C_ 56.10, and the downfield shift of C-7 to δ_C_ 165.78 was assigned to a methoxyl carbon at position 7. This data confirms the structure of metabolite 4 as a herbacetin 7-methyl ether (Hussein et al. 1997). In addition, the presence of a signal at δ_C_ 102.27 indicates an anomeric proton of hexose. Furthermore, the signal at δ_C_ 61.06 was assigned to the unsubstituted C-6`` of glucose unit. The ^13^C NMR investigations and literature data were used to characterize the glucose unit, which revealed that it is a glucopyranoside (Mizuno et al. 2022). An explanation that is consistent with the chemical shift values previously mentioned indicates metabolite 4 to be herbacetin 7-methyl ether-3-*O-β-D*-glucopyranoside.

Flavonol glycosides have both antioxidant and antimicrobial traits. They control reactive oxygen species (ROS) accumulation by scavenging ROS, contributing to their antioxidant activity, which is crucial for plants under various environmental stressors (Dias et al. 2021; Abu El Wafa et al. 2023). So, the antioxidant effect of the isolated metabolites was assessed using various assays, ABTS revealed that metabolites 4, 2, and 3 exhibit significant antioxidant potential, with IC_50_ values of 12.23 ± 0.08900, 13.90 ± 0.1400, and 7.92 ± 0.1451 µg/ml, respectively. Metabolite 1, with the lowest IC_50_ value of 6.993 ± 0.05999 µg/mL, outperforms the others with the highest scavenging activity and FRAP ability. While all metabolites show some degree of power reduction, metabolite 1 also has the highest antioxidant capacity. Metabolites 2 and 4 display moderate activity, while metabolite 3 has the least. Metabolite 1, which has emerged as the most potent antioxidant, is a promising candidate for studying anti-MRSA activity. This is in comparison to the total extract of the *C. madurensis* aerial part. Previous research has shown that alcoholic extracts from the stems and flowers of *C. madurensis* exhibit stronger antimicrobial effects against *S. aureus, P. aeruginosa*, and *E. coli* than leaf extracts and total saponins. The variation in antibacterial activity among the extracts could be attributed to differences in their composition, the structure of their active ingredients, and the way these ingredients interact with the bacterial cell wall (Ibrahim et al. 2017). The influence of flavonoids on cell membranes implies that they can obstruct fatty acid synthesis through enzymatic pathways, leading to the inhibition of cell membrane synthesis. Additionally, flavonoids can hinder the creation of various virulence factors that affect motility, bacterial attachment to the host, and biofilm production (Gallegos et al. 2016) illustrated in (Figure S15, supplementary data).

The relationship between the antioxidant and antimicrobial activities of flavonol glycosides, such as metabolite 1, is complex and can vary depending on the specific compound and the organism it interacts with. Both these properties contribute to their overall health benefits (Tagousop et al. 2018). However, a comprehensive understanding of the interplay between these activities requires further research. This could potentially lead to the development of more effective treatments for MRSA infections. Methicillin-resistant *S. aureus* (MRSA) is an infection that poses a treatment challenge due to its resistance to antibiotics. While Staphylococcus bacteria are typically harmless, they can lead to severe diseases that can be fatal (Howden et al. 2023). *S. aureus* is a bacterial species that resides in humans and can cause a range of clinical symptoms (Dayan et al. 2016). If *S. aureus* enters the body through a skin cut, it can lead to infections ranging from mild to severe, which can be fatal in some instances. A high incidence of skin infection has been recognized as a risk factor for MRSA nasal carriage. Moreover, patients with pathological skin conditions are more prone to disseminate infectious strains (Gordon and Lowy 2008). Antibiotic susceptibility tests are key to finding effective treatments for bacterial infections like MRSA. Most antibiotics tested showed potential effectiveness against MRSA (Qodrati et al. 2022). However, the choice of treatment depends on various factors including the patient’s health, antibiotic side effects, and infection severity. Antibiotic resistance is a major concern, so even if an antibiotic inhibits MRSA in a lab, it must be used wisely in clinical settings to prevent resistance (Dadgostar 2019). The antibiotics, belonging to different groups and operating via different mechanisms, were either susceptible or resistant to MRSA. The inhibition zones’ diameters were ranked in descending order as follows: Levofloxacin LEV-5, Rifaximin RF-30, Streptomycin S-10, and Cefuroxime CXM-30. However, MRSA showed resistance to Ampicillin/Sulbactam SAM-20. Antimicrobial agents are commonly used to treat microbial infections caused by medical conditions, environmental factors, or food poisoning, with MRSA infection being a key example (Enright et al. 2002). Recently, plant flavonoids have attracted significant interest due to their potential to fight pathogenic microbes (Górniak et al. 2019). This encourages us to examine the antibacterial effects of the *C. madurensis* plant extract and metabolite 1 using the well agar diffusion method. The plant extract and metabolite 1 were tested against MRSA at concentrations of 1.0, 0.5, and 0.25 mg/ml. As shown in Fig. 6, The plant extract and metabolite 1 were both tested for their antibacterial activity against MRSA using a viable cell count test. The plant extract showed substantial antibacterial activity at concentrations of 1.0 and 0.5 mg/ml. However, metabolite 1 demonstrated the most potent antibacterial effect against MRSA at all tested concentrations. Bacterial inhibition was observed within a range of dilutions, with complete inhibition occurring at 10–5 dilutions for 1.0 mg/ml of metabolite 1. As the treatment concentrations increased, the CFU/ml values decreased, indicating a reduction in bacterial viability (Thieme et al. 2021). Metabolite 1 resulted in the lowest CFU/ml at a concentration of 1.0 mg/ml, suggesting it was more effective at inhibiting MRSA growth compared to the plant extract.

Gamma radiation showed a higher total flavonoid content than non-irradiated plants. However, the effects of gamma radiation can vary greatly depending on factors like the plant type, radiation dose, and specific flavonoid glycoside (Alivandi Farkhad and Hosseini 2020). In certain instances, components such as catechin and kaempferol flavonoid glycosides have been observed to notably decrease with gamma radiation (Moghaddam et al. 2011; Breitfellner et al. 2002). The study assessed the effects of gamma irradiation on the antibacterial activity of *C. madurensis* plant extract and metabolite 1 against MRSA. Metabolite 1 was irradiated at 50 and 100 Gy. The results showed that gamma irradiation did not enhance the inhibitory effect of the substances on MRSA compared to un-irradiated samples. However, the most significant effect was observed at 100 Gy and a concentration of 1.0 mg/ml for both tested samples, with larger inhibition zone diameters indicating stronger antibacterial activity.

FTIR spectroscopy, a crucial tool in antimicrobial research, aids in understanding the molecular interactions between antimicrobial agents and microorganisms and is instrumental in the rapid identification of antibiotic-resistant bacteria (Faghihzadeh et al. 2016; Wang-Wang et al. 2022). Bacterial cells are composed of proteins, fatty acids, carbohydrates, nucleic acids, and lipopolysaccharides and these biochemical molecules can be assigned by FT-IR spectra (Yang et al. 2023). The commonly used region for infrared absorption spectroscopy is 4000 ~ 400 cm^−1^ because the absorption radiation of most organic compounds and inorganic ions is within this region (Yang et al. 2023). This was exemplified in a study that used FTIR spectroscopy to analyze the spectral changes in MRSA filtrates, both untreated and treated with a plant extract and metabolite 1. The research identified significant disparities between the control and treated spectra. The treatment notably diminished transmittance (%), suggesting a reduction in essential bacterial cell components. This implies that the treatment may have modified the molecular structure of the MRSA cells, potentially impacting their viability. The analysis unveiled considerable differences across the entire spectral range, especially in the membrane amphiphile region (3000–2800 cm^−1^), the phospholipids DNA-RNA regions (1200 to 1500 cm^−1^), the proteins and amides I and II regions (1500 to 1700 cm^−1^), the polysaccharides vibrations regions (900 to 1200 cm^−1^), and the fingerprint region (600 to 900 cm^−1^) (Preisner et al. 2010). A vibration band at 1628 cm^−1^, representative of amide I of the protein’s alpha-helical structure, was significantly diminished while amide II at 1550 cm^−1^, and amide III at 1260 cm^−1^. The robust and broadband at 3300 cm^−1^ could be ascribed to the N–H stretching vibration of nucleic acid constituents such as adenine, cytosine, and/or guanine, and possibly to OH groups potentially bound to nucleic acid (Filip et al. 2009). In addition, in each acyl chain, minimize the CH stretching band intensity value at 2354.92 cm^−1^ in the spectrum of the treated bacteria indicating a lowering in the methyl group numbers compared to untreated bacteria. Furthermore, in the spectrum of treated bacteria, a decrease in the intensity of the stretching bands indicates a reduction in the number of functional groups compared to untreated bacteria that in turn reflect the role of plant extract and metabolite 1 in reducing the bacterial growth (Kamnev et al. 2002).

Through the process of docking, we can evaluate the antioxidant and antimicrobial properties of molecules by comparing their binding energies with those of known activators or inhibitors. This technique also aids in the development of new molecules with enhanced antioxidant and antimicrobial properties by modifying their chemical structures or adding functional groups. Docking is instrumental in predicting the binding affinity and mode of action of potential drug candidates, and in identifying new targets for drug design. It is particularly useful in studying the antioxidant and antimicrobial activities of molecules, which are key attributes for a multitude of biological processes and diseases (Al-Khaldi et al. 2021). The objective of this study was to elucidate the potential antioxidant mechanisms of isolated metabolites (1–4). Docking simulations were employed to assess the affinity between four metabolites and the binding sites of arachidonate-5-lipoxygenase (PDB: 6n2w) (Ley-Martínez et al. 2022). Due to its inhibitory activity on 5-lipoxygenase (Kahnt et al. 2022), NDGA was chosen as a reference co-ligand for the docking study. The docking scores indicated that all four metabolites had lower binding energy values compared to the co-crystal ligand. Lipoxygenases (LOX), enzymes that catalyze lipid oxidation, are recognized for their role in generating oxidized lipids within atherosclerotic plaques. The enzyme arachidonate 5-lipoxygenase (5-LOX) plays a pivotal role in the synthesis of leukotrienes, and its inhibition provides cellular protection against oxidative stress (Ley-Martínez et al. 2022). The active site of 5-LOX comprises amino acid residues such as Gln557, His372, Gln363, Leu673, Ala410, His432, His-367, His600, and His550 (Li et al. 2011). Four chromen-4-one compounds exhibited a higher docking score of over -12 kcal/mol, compared to the co-crystallized ligand (NDGA) with a score of -11.01 kcal/mol, suggesting a robust affinity for the 5-LOX enzyme. The NDGA molecule establishes four hydrogen bonds with the amino acid residues Ile 406, His 372, His 600, and Arg 596, and forms four hydrophobic contacts with Isoleucine 673, Phenylalanine 359, Alanine 603, and Alanine 410 (Figure S16, Table S1 supplementary data).

The four metabolites (1–4) demonstrated a similar binding mode within the 5-LOX active site to NDGA (Figures S3-S6). Their calculated binding energies were -14.00, -13.54, -13.34, and -12.32 kcal/mol, respectively. The chromen 4-one moiety of the compounds play a significant role in the binding within the pocket, generating hydrogen bonds, hydrophobic interactions, or both. Gossypetin 8-methoxy,3-*O*-β-D-4C1-xylopyranoside (metabolite 1), the compound with the highest docking score, exhibited twelve hydrogen bonds and formed two Pi-Pi T-shaped bonds with Phe359 and Trp599, and a Pi-Alkyl bond with Ala603 (Figure S17). Gossypetin-8-*O*-β-D-glucopyranoside (metabolite 2) engaged in hydrogen bonding interactions with several amino acid residues and exhibited four Pi-Pi T-shaped contacts and a Pi-Alkyl bond with Ala603 (Figure S18). Metabolite 3; Kaempferol 3-*O*-ß–D-glucoside’s chromen-4-one engaged in four hydrophobic contacts and formed five hydrogen bonds with His367, Gln363, and His600. A Pi-Pi T-shaped interaction was observed between the phenolic ring and Try599 (Figure S19). Herbacetin 7-methyl ether-3-*O*-β-D-glucopyranoside (metabolite 4) had eight hydrogen connections and displayed a Pi-Pi T-shaped interaction with Try599 and two Pi-Alkyl interactions (Figure S6). From the aforementioned results, metabolite 1 exhibited the highest score, leading to its selection for further analysis. The antimicrobial effectiveness of this metabolite implies that its mode of action might involve interactions with key enzymes that play a role in eliminating bacteria. These enzymes include dihydrofolate reductase (PDB ID: 3sr5) (Li et al. 2011), DNA gyrase (PDB ID: 3g75) (Ronkin et al. 2010), penicillin-binding protein (PBP2a) (PDB ID: 1mwt) (Lim and Strynadka 2002), and threonyl-tRNA Synthetase (PDB ID: 1nyq) (Torres-Larios et al. 2003). The co-crystallized ligands were re-docked into their respective enzymes, resulting in a root-mean-square deviation (RMSD) of 0.62 Å (Lipoxygenase enzyme), 0.12 Å (dihydrofolate reductase), 0.08 Å (DNA gyrase), 0.70 Å (PBP2a), and 0.65 Å (threonyl-tRNA synthetase) between the docked and co-crystallized ligand. This outcome validates the docking methodology employed. The study concluded that hydrogen bonds, hydrophobic interactions, and Pi–Pi interactions were the most significant interactions. **Dihydrofolate reductase (DHFR**) is an enzyme that plays a pivotal role in converting dihydrofolate into tetrahydrofolate. This conversion process is essential for the synthesis of purines, thymidylate, and certain amino acids. DHFR is a significant target in a variety of therapeutic areas, including the treatment of cancer and the development of anti-infective drugs.

The co-crystal ligand Q12 establishes three hydrogen bonds with vital residues and demonstrates π-π stacking with the Phe93 residue. Additionally, it forms seven Pi-Alkyl bonds (refer to Figure S7 and Table S2). Gossypetin 8-methoxy 3-*O*-β-D-xylopyranoside (also known as metabolite 1), with a docking score of -19.56 kcal/mol, exhibits a stronger affinity compared to Q21, which has a score of -12.28 kcal/mol. Metabolite 1 forms ten hydrogen bonds, displays two instances of π–π stacking, and has five Pi-Alkyl interactions (refer to Figure S8 and Table S2). In the ATP-binding pocket of **DNA gyrase B**, the compound gossypetin 8-methoxy, 3-*O*-β-D-xylopyranoside successfully attached to the ATP binding sites of the *Staphylococcus aureus* gyrase B enzyme. It utilized the same binding strategy as the cocrystal ligand (B48) and achieved a favorable docking score of -15.39 kcal/mol, surpassing B48’s score of -10.51 kcal/mol.

The binding mechanism of B48 involved a single hydrogen bond with the crucial amino acid Asp81 and three pi-interactions with Ile86 (refer to Figure S9). Conversely, gossypetin 8-methoxy, 3-*O*-β-D-xylopyranoside formed hydrogen bonds with Thr173, Asn54, and Asp81 via its sugar component. Its chromen-5-one ring established hydrogen bonds with Ser129 and Asp57 and participated in three Pi-Alkyl interactions with Ile86 and Ile102 (refer to Figure S10 and supplementary data).

A docking analysis was conducted to comprehend the interaction between gossypetin 8-methoxy, 3-*O*-β-D-xylopyranoside and the binding site of the Penicillin-Binding Protein 2a (PBP2a) enzyme in *Staphylococcus aureus*. The co-crystal ligand (PNM) demonstrated significant interactions with specific amino acids within the active site groove, achieving a docking score of -14.18 kcal/mol. It established hydrogen bonds with residues Ser462, Ser598, Ser403, Asn 464, Arg445, and Thr600, and interacted with Met 641 through Pi-sulfur and alkyl bonds, and with Lys597, Lys406, and Lys597 through attractive charge (refer to Figure S11 and Table S2). Gossypetin 8-methoxy, 3-*O*-β-D-xylopyranoside, on the other hand, achieved a higher docking score of -20.67 kcal/mol. Its chromen-4one moiety formed a Pi-donor hydrogen bond with Thr600, conventional hydrogen bonds with Ser403, Ser462, Ser598, and Met641, carbon-hydrogen bonds with Asn464 and Ser462, a Pi-Alkyl bond with Ala642, and one sulfur bond and two Pi-sulfur bonds with Met641. The sugar ring established two hydrogen connections with Gln613 and Glu602, and the phenolic ring formed three hydrogen bonds with Thr444 and Asn464 (refer to Figure S12 and Table S2). In the active site of Threonyl-tRNA Synthetase (ThrRS), aminoacyl-tRNA synthetases, which facilitate the attachment of amino acids to their corresponding transfer RNAs, are potential targets for the development of antibiotics due to their integral role in protein synthesis and the translation of the genetic code. The co-crystal ligand (TSB) and gossypetin 8-methoxy, 3-*O*-β-D-xylopyranoside interact with the amino acid residues in the threonyl-binding region. They have docking scores of -18.46 and -19.15 kcal/mol, respectively. TSB forms hydrogen bonds with several amino acids, a Metal-Acceptor link with a zinc atom, and Pi-interactions with Asp385, Leu383, and Met334 residues (refer to Figure S13 and Table S2). Gossypetin 8-methoxy, 3-*O*-β-D-xylopyranoside demonstrates a similar binding mode, interacting with the adenine- and threonyl-binding regions. It forms hydrogen bonds with Arg518, Ser522, Lys471, and Gly519, a Metal-Acceptor bond with two zinc atoms, and engages in Pi-interactions with Leu383 and Met334 (refer to Figure S14 and Table S2).

A common correlation is often observed between antioxidants and antibacterial properties in various natural extracts. The structure of these antioxidants plays a crucial role in establishing this correlation. Gossypetin 8-methoxy, 3-O-β-D-xylopyranoside (metabolite 1), a flavonol glycoside of *C. madurensis*, exhibits significant antioxidant properties. Therefore, the antioxidant properties of metabolite 1 could potentially contribute to its effectiveness against methicillin-resistant *Staphylococcus aureus* (MRSA) ATCC 6538. Both the plant extract and metabolite 1 demonstrate potential antibacterial properties against MRSA, with metabolite 1 showing superior antibacterial activity at all tested concentrations. These findings underscore the potential of plant-derived metabolites in combating antibiotic-resistant bacteria like MRSA. Gamma radiation can significantly affect plant extracts and metabolites, potentially enhancing their medicinal properties. However, the exact impacts can vary greatly depending on numerous factors. While gamma irradiation did not enhance the overall antibacterial activity of the plant extract and metabolite 1 against MRSA, it appeared to have some effect at specific doses and concentrations.

The FTIR study offers valuable insights into the potential antibacterial mechanism of the plant extract and metabolite 1 against MRSA. However, additional studies are needed to fully understand these mechanisms and their implications for the development of new antibacterial agents. Docking analysis was conducted to understand the interaction between metabolite 1 and the binding site of the Penicillin-Binding Protein 2a enzyme in *Staphylococcus aureus*. This study suggests that the antimicrobial effectiveness of a metabolite could be due to its interactions with key enzymes involved in bacterial eradication. These enzymes include dihydrofolate reductase, DNA gyrase, penicillin-binding protein, and threonyl-tRNA Synthetase. The docking methodology used in the study was validated by the close match between the docked and co-crystallized ligands. Gossypetin 8-methoxy 3-*O*-β-D-xylopyranoside achieved a higher docking score and formed several bonds and interactions. In conclusion, additional research is necessary to validate these results, fully comprehend these effects, and explore their potential clinical applications.

## Electronic supplementary material

Supplementary Material 1

## Data Availability

The datasets used and/or analyzed during the current study are available from the corresponding author on reasonable request. nohaseifeldein@azhar.edu.eg.

## Abbreviations

MRSA: Methicillin-resistant *Staphylococcus aureus*
MDR: Multidrug resistance
*S. aureus*: *Staphylococcus aureus*
TAC: Total antioxidant capacity
FRAP: Ferric reducing antioxidant power
ABTS: 2,2′-Azino-bis (3-ethylbenzothiazoline-6-sulfonic acid)
NCRRT: National center for radiation research and technology
NB: Nutrient broth medium
ZOI: Zone of Inhibition
MIC: Minimum inhibitory concentration
CFU/ml: Colony-forming units per millimeter
FTIR: Fourier-transform infrared spectra
5-LOX: Arachidonate 5-lipoxygenase
NDGA: Nordihydroguaiaretic acid
DHFR: Dihydrofolate reductase
PBP2a: Penicillin-binding protein 2a
ThrRS: Threonyl-tRNA synthetase
