# Supplementary material for: Crotalaria madurensis flavonol glycosides’ antibacterial activity against Staphylococcus aureus
Source: AMB Express. 2024 Nov 4;14:118. doi: 10.1186/s13568-024-01776-3 (PMC11535145; doi:10.1186/s13568-024-01776-3)
Supplement: Supplementary file 1 — Supplementary Material 1 [file 13568_2024_1776_MOESM1_ESM.docx]

**Supplementary materials**

***Crotalaria madurensis* flavonol glycosides' antibacterial activity against**

***Staphylococcus aureus***

**Hala Sh. Mohammed^1^, Salwa A. Abu El wafa^1^, Mona H. Ibrahim^2^, Rasha Mohammad Fathy^3^, Noha A. Seif-Eldein^1^**

^1^ Pharmacognosy and Medicinal Plants Department, Faculty of Pharmacy, Al-Azhar University, Cairo, Egypt.

^2^ Department of Pharmaceutical Medicinal Chemistry and Drug Design, Faculty of Pharmacy (Girls), Al-Azhar University, Cairo 11884, Egypt.

# ^3^ Drug Radiation Research Department, National Center for Radiation Research and Technology (NCRRT), Egyptian Atomic Energy Authority, Cairo, Egypt.

|  | **Contents** | **Page** |  |
| --- | --- | --- | --- |
| **Table S1** | Binding energy, bond type, amino acids and distance of bonds obtained from the docking calculations of chromen-4-one’s metabolites with Lipoxygenase enzyme | **2** |  |
| **Table S2** | Binding energy, bond type, amino acids and distance of bonds obtained from the docking calculations of **Gossypetin 8-methoxy, 3-*O*-β-D-^4^C_1_-xylopyranoside** with dihydrofolate reductase, DNA gyrase, Penicillin binding protein (PBP2a) and Threonyl-tRNA Synthetase enzymes. | **3** |  |
| **Figure S1** | Flavonoids inhibit the mechanism of bacterial cells in MRSA through various methods, depicted diagrammatically. | **5** |  |
| **Figure S2** | 2D (left) and 3D (right) Binding mode of co-crystal ligand (**NDGA**) in the active site of 5-LOX enzyme. | **6** |  |
| **Figure S3** | 2D (left) and 3D (right) Binding mode of **gossypetin 8-methoxy, 3-*O*-*β*-*D*-xylopyranoside** (metabolite 1) in the active site of 5-LOX enzyme. |  |  |
| **Figure S4** | 2D (left) and 3D (right) Binding mode of **gossypetin 8-*O* *β*-*D* – glucopyranoside** (metabolite 2) in the active site of 5-LOX enzyme. | **7** |  |
| **Figure S5** | 2D (left) and 3D (right) Binding mode of **kaempferol 3-O*-ß–D*-glucoside** (metabolite 3) in the active site of 5-LOX enzyme. |  |  |
| **Figure S6** | 2D (left) and 3D (right) Binding mode of **herbacetin-7-methyl ether-3-O*-β-D*-glucopyranoside** (metabolite 4) in the active site of 5-LOX enzyme. | **8** |  |
| **Figure S7** | 2D (left) and 3D (right) Binding mode of **Q21** in the active site of DHFR enzyme. |  |  |
| **Figure S8** | 2D (left) and 3D (right) Binding mode of **gossypetin 8-methoxy, 3-O-*β*-*D*-^4^C1-xylopyranoside** in the active site of DHFR enzyme. | **9** |  |
| **Figure S9** | 2D (left) and 3D (right) Binding mode of co-crystal ligand ([B48](https://www.rcsb.org/ligand/B48)) into the ATP binding site of DNA gyrase B enzyme. |  |  |
| **Figure S10** | 2D (left) and 3D (right) Binding mode of **gossypetin 8-methoxy, 3-O-*β*-*D*-xylopyranoside** into the ATP binding site of DNA gyrase B enzyme. | **10** |  |
| **Figure S11** | 2D (left) and 3D (right) Binding mode of co-crystal ligand ([PNM](https://www.rcsb.org/ligand/PNM)) into PBP2a enzyme's binding site. |  |  |
| **Figure S12** | 2D (left) and 3D (right) Binding mode **gossypetin 8-methoxy, 3-O-*β*-*D*-^4^C1-xylopyranoside** into PBP2a enzyme's binding site. | **11** | |
| **Figure S13** | 2D (left) and 3D (right) Binding mode of co-crystal ligand into ThrRS enzyme's binding site. |  |  |
| **Figure S14** | 2D (left) and 3D (right) Binding mode **gossypetin 8-methoxy 3-*O*-*β*-*D*-xylopyranoside** into ThrRS enzyme's binding site. | **12** | |
| **Figure S15** | The root means square deviation between the original and docked poses of the cocrystal ligands of Lipoxygenase enzyme (PDB: 6n2w) was 0.62 Å. |  |  |
| **Figure S16** | The root means square deviation between the original and docked poses of the cocrystal ligands of **dihydrofolate reductase** enzyme (PDB: 3sr5) was 0.12 Å. | **13** |  |
| **Figure S17** | The root means square deviation between the original and docked poses of the cocrystal ligands of **DNA gyrase** enzyme (PDB: 3g75) was 0.08 Å. |  |  |
| **Figure S18** | The root means square deviation between the original and docked poses of the cocrystal ligand ([PNM](https://www.rcsb.org/ligand/PNM)) of **Penicillin binding protein (PBP2a) enzyme (PDB: 1mwt)** was 0.70 Å. | **14** |  |
| **Figure S19** | The root means square deviation between the original and docked poses of the cocrystal ligands of **threonyl-tRNA Synthetase (PDB: 1nyq)** was 0.65 Å. |  |  |

**Table S1:** Binding energy, bond type, amino acids and distance of bonds obtained from the docking calculations of chromen-4-one’s metabolites with Lipoxygenase enzyme.

| **Lipoxygenase (PDB: 6n2w)** | | |
| --- | --- | --- |
| **Compound** | **Amino acid/ type of interaction/distance Å** | **Binding energy kcal/mol** |
| **Ligand** | His372/ Conventional Hydrogen Bond/2.93 Å  Arg596/ Conventional Hydrogen Bond/3.08 Å  His600/ Conventional Hydrogen Bond/3.10 Å  Ile406/ Conventional Hydrogen Bond/3.37 Å  Ile673/ Pi-Anion/4.68 Å  Phe359/ Pi-Pi T-shaped/5.11 Å  Ala603/ Alkyl/4.39 Å  Ala410/ Pi-Alkyl/4.63 Å | -11.01 |
| **Gossypetin 8-methoxy, 3-*O*-β-D-^4^C_1_-xylopyranoside** | Gln557/ Conventional Hydrogen Bond/2.53 Å  Arg596/ Conventional Hydrogen Bond/1.97 Å  Gln363/ Conventional Hydrogen Bond/3.17 Å  Gln363/ Conventional Hydrogen Bond/3.26 Å  His367/ Conventional Hydrogen Bond/3.17 Å  Thr364/ Conventional Hydrogen Bond/2.94 Å  His600/ Conventional Hydrogen Bond/2.86 Å  Gln363/ Carbon Hydrogen Bond/2.96 Å  Thr364/ Carbon Hydrogen Bond/2.79 Å  His600/ Carbon Hydrogen Bond/2.95 Å  Gln363 Carbon Hydrogen Bond/3.63 Å  His367/ Pi-Donor Hydrogen Bond/2.60 Å  Phe359/ Pi-Pi T-shaped/5.27 Å  Trp599/ Pi-Pi T-shaped/5.09 Å  Ala603/ Pi-Alkyl/4.39 Å | -14.00 |
| **Gossypetin - 8-*O* β-D –^4^C_1_- glucopyranoside** | Gln557/ Conventional Hydrogen Bond/2.85 Å  Arg596/ Conventional Hydrogen Bond/2.40 Å  Arg596/ Conventional Hydrogen Bond/2.73 Å  Arg596/ Conventional Hydrogen Bond/2.98 Å  Arg596/ Conventional Hydrogen Bond/1.87 Å  Gln363/ Conventional Hydrogen Bond/3.09 Å  His600/ Conventional Hydrogen Bond/2.81 Å  Gln363/ Conventional Hydrogen Bond/2.34 Å  His600/ Carbon Hydrogen Bond/2.85 Å  Phe359/ Pi-Pi T-shaped/5.19 Å  His432/ Pi-Pi T-shaped/4.33 Å  Trp599/ Pi-Pi T-shaped/5.79 Å  Trp599/ Pi-Pi T-shaped/5.39 Å  Ala603/ Pi-Alkyl/4.50 Å | -13.54 |
| **Kaempferol 3-*O-ß–D*-glucoside (Astragalin)** | His367/ Conventional Hydrogen Bond/2.06 Å  Gln363/ Conventional Hydrogen Bond/3.23 Å  Gln363/ Conventional Hydrogen Bond/2.88 Å  His367/ Conventional Hydrogen Bond/3.27 Å  His600/ Conventional Hydrogen Bond/3.33 Å  His367/ Pi-Donor Hydrogen Bond/3.82 Å  Leu607/ Pi-Sigma/2.48 Å  Trp599/ Pi-Pi T-shaped/5.04 Å  Ala603/ Pi-Alkyl/4.74 Å  Ala603/ Pi-Alkyl/4.21 Å  Ala603/ Pi-Alkyl/5.19 Å  Leu607/ Pi-Alkyl/4.64 Å | -13.43 |
| **Herbacetin-7-methyl ether-3-*O-β-D*-glucopyranoside** | His372/ Conventional Hydrogen Bond/2.11 Å  Arg596/ Conventional Hydrogen Bond/2.75 Å  Ile673/ Conventional Hydrogen Bond/3.31 Å  Asn407/ Conventional Hydrogen Bond/3.32 Å  Ala410/ Conventional Hydrogen Bond/2.89 Å  His367/ Carbon Hydrogen Bond/2.37 Å  His372/ Carbon Hydrogen Bond/2.87 Å  Pro569/ Carbon Hydrogen Bond/3.37 Å  Trp599/ Pi-Pi T-shaped/5.14 Å  Ala410/ Pi-Alkyl/5.44 Å  Leu414/ Pi-Alkyl/4.62 Å | -12.32 |

**Table S2:** Binding energy, bond type, amino acids and distance of bonds obtained from the docking calculations of **Gossypetin 8-methoxy, 3-*O*-β-D-^4^C_1_-xylopyranoside** with dihydrofolate reductase, DNA gyrase, Penicillin binding protein (PBP2a) and Threonyl-tRNA Synthetase enzymes.

|  | **Co-crystal ligand** | | | **Gossypetin 8-methoxy, 3-*O*-β-D-^4^C_1_-xylopyranoside** | | |
| --- | --- | --- | --- | --- | --- | --- |
| **Dihydrofolate reductase (PDB ID: 3sr5)** | (Amino acid/ type of interaction/distance) | Binding energy kcal/mol | | Amino acid/ type of interaction/distance | Binding energy kcal/mol | |
|  | Asp28/ Salt Bridge/2.93 Å  Leu6/ Conventional Hydrogen Bond/2.85 Å  Asp28/ Conventional Hydrogen Bond/2.58 Å  Val7/ Carbon Hydrogen Bond/2.53 Å  Phe93/ Pi-Donor Hydrogen Bond/4.15 Å  Phe93/ Pi-Pi Stacked/4.63 Å  Ile51/Alkyl/4.39 Å  Leu6/Pi-Alkyl/5.38 Å  Ala8/Pi-Alkyl/4.10 Å  Val32/Pi-Alkyl/5.02 Å  Leu21/Pi-Alkyl/5.19 Å  Val32//Pi-Alkyl/5.10 Å  Leu29/Pi-Alkyl/4.58 Å  Ile51/Pi-Alkyl/4.84 Å | -12.28 | | Ser50/ Conventional Hydrogen Bond/2.06 Å  Ser50/ Conventional Hydrogen Bond/2.02 Å  Thr122/ Conventional Hydrogen Bond/2.57 Å  Phe93/ Conventional Hydrogen Bond/2.90 Å  Asp28/ Conventional Hydrogen Bond/2.94 Å  Gly16/ Carbon Hydrogen Bond/2.23 Å  Thr47/ Carbon Hydrogen Bond/2.24 Å  Ser50/ Carbon Hydrogen Bond/3.01 Å  Gly95/ Carbon Hydrogen Bond/2.43 Å  Ile15/ Carbon Hydrogen Bond/2.77 Å  Phe93/Pi-Pi Stacked/3.97 Å  Phe93/Pi-Pi Stacked/3.93 Å  Leu21/Pi-Alkyl/4.59 Å  Leu21/Pi-Alkyl/5.13 Å  Ala8/Pi-Alkyl/4.93 Å  Leu21/Pi-Alkyl/5.02 Å  Val32/Pi-Alkyl/5.388 Å | -19.56 | |
|  | Co-crystal ligand ([B48](https://www.rcsb.org/ligand/B48)) | | | **Gossypetin 8-methoxy, 3-*O*-β-D-^4^C_1_-xylopyranoside** | | |
| **DNA gyrase (PDB ID: 3g75)** | Amino acid/ type of interaction/distance | Binding energy kcal/mol | | Amino acid/ type of interaction/distance | Binding energy kcal/mol | |
|  | Asp81/ Conventional Hydrogen Bond/2.03 Å  Arg84/ Pi-Cation/4.06 Å  Ile86/ Alkyl/4.21 Å  Ile102/ Alkyl/4.89 Å  Ile86/ Pi-Alkyl/5.23 Å  Ile86/ Pi-Alkyl/4.45 Å  Pro87/ Pi-Alkyl/4.23 Å | -10.51 | | Thr173/ Conventional Hydrogen Bond/2.88 Å  Asn54/ Conventional Hydrogen Bond/2.66 Å  Asp81/ Conventional Hydrogen Bond/1.73 Å  Asp81/ Conventional Hydrogen Bond/2.16 Å  Ser129/ Carbon Hydrogen Bond/2.93 Å  Asp81/ Carbon Hydrogen Bond/2.55 Å  Asp57/ Carbon Hydrogen Bond/3.00 Å  Ile86/ Pi-Alkyl/4.48 Å  Ile102/ Pi-Alkyl/4.92 Å  Ile86/ Pi-Alkyl/4.48 Å  Ile102/ Pi-Alkyl/4.55 Å | -15.39 | |
|  | Co-crystal ligand ([PNM](https://www.rcsb.org/ligand/PNM)) | | | **Gossypetin 8-methoxy, 3-*O*-β-D-^4^C_1_-xylopyranoside** | | |
| **Penicillin binding protein (PBP2a) (PDB ID: 1mwt)** | Amino acid/ type of interaction/distance | | Binding energy kcal/mol | Amino acid/ type of interaction/distance | | Binding energy kcal/mol |
|  | Lys597/ Salt Bridge;Attractive Charge/2.26 Å  Lys406/ Attractive Charge/5.57 Å  Lys597/ Attractive Charge/4.55 Å  Ser462/ Conventional Hydrogen Bond/2.27 Å  Ser462/ Conventional Hydrogen Bond/2.36 Å  Ser598/ Conventional Hydrogen Bond/1.83 Å  Thr600/ Conventional Hydrogen Bond/1.99 Å  Ser403/ Conventional Hydrogen Bond/2.07 Å  Arg445/ Carbon Hydrogen Bond/2.80 Å  Ser598/ Carbon Hydrogen Bond/2.60 Å  Thr600/ Carbon Hydrogen Bond/2.30 Å  Asn464/ Carbon Hydrogen Bond /2.51 Å  Met641/ Pi-Sulfur/5.10 Å  Met641/ Alkyl/3.71 Å  Tyr446/ Pi-Alkyl/4.37 Å | | -14.18 | Ser403/ Conventional Hydrogen Bond/2.68 Å  Thr444/ Conventional Hydrogen Bond/2.75 Å  Ser462/ Conventional Hydrogen Bond/2.77 Å  Ser598/ Conventional Hydrogen Bond/2.95 Å  Gln613/ Conventional Hydrogen Bond/2.94 Å  Glu602/ Conventional Hydrogen Bond/2.88 Å  Thr444/ Conventional Hydrogen Bond/1.91 Å  Met641/ Conventional Hydrogen Bond/2.03 Å  Ser462/ Conventional Hydrogen Bond/2.51 Å  Asn464/ Carbon Hydrogen Bond/2.58 Å  Ser462/ Carbon Hydrogen Bond/2.32 Å  Asn464/ Carbon Hydrogen Bond/2.67 Å  Asn464/ Carbon Hydrogen Bond/2.38 Å  Met641/ Sulfur-X/2.82 Å  Asn464/ Pi-Donor Hydrogen Bond/3.38 Å  Thr600/ Pi-Donor Hydrogen Bond/3.65 Å  Met641/ Pi-Sulfur/4.77 Å  Met641/ Pi-Sulfur/5.11 Å  Ala642/ Pi-Alkyl/5.41 Å | | -20.67 |
|  | Co-crystal ligand | | | **Gossypetin 8-methoxy, 3-*O*-β-D-^4^C_1_-xylopyranoside** | | |
| **Threonyl-tRNA Synthetase (PDB ID: 1nyq)** | Amino acid/ type of interaction/distance | | Binding energy kcal/mol | Amino acid/ type of interaction/distance | Binding energy kcal/mol | |
|  | Arg365/ Conventional Hydrogen Bond/1.91  Arg365/ Conventional Hydrogen Bond/2.39  Lys471/ Conventional Hydrogen Bond/1.94  Lys471/ Conventional Hydrogen Bond/2.07  Thr523/ Conventional Hydrogen Bond/2.47  Arg526/ Conventional Hydrogen Bond/1.98  Arg518/ Conventional Hydrogen Bond/1.88  Asp385/ Conventional Hydrogen Bond/2.33  Ser386/ Conventional Hydrogen Bond/2.81  Gly519/ Carbon Hydrogen Bond/2.96  Ser522/ Carbon Hydrogen Bond/2.93  Arg526/ Carbon Hydrogen Bond/2.61  Arg526/ Carbon Hydrogen Bond/2.34  Gln490/ Carbon Hydrogen Bond/2.50  ZN1001/ Metal-Acceptor/2.48  Asp385/ Pi-Anion/3.76  Leu383/ Pi-Sigma/2.55  Met334/ Pi-Sulfur/5.52  Met334/ Pi-Sulfur/4.27  Cys336/ Pi-Sulfur/4.72 | | -18.46 | Lys471/ Conventional Hydrogen Bond/2.05  Arg518/ Conventional Hydrogen Bond/2.84  Ser522/ Conventional Hydrogen Bond/2.44  Lys471/ Carbon Hydrogen Bond/2.33  Gly519/ Carbon Hydrogen Bond/2.91  Gly519/ Carbon Hydrogen Bond/2.51  ZN1001/ Metal-Acceptor/3.19  ZN1001/ Metal-Acceptor/2.47  Leu383/ Pi-Sigma/2.47  Met334/ Pi-Sulfur/5.09  Leu383/ Pi-Alkyl/5.27 | -19.15 | |


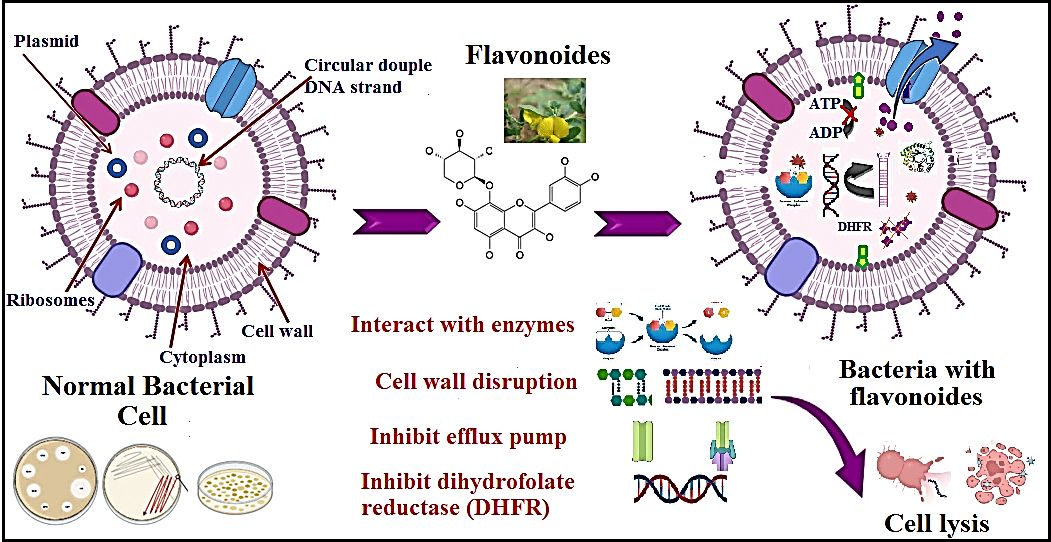


**Figure S1.** Flavonoids inhibit the mechanism of bacterial cells in MRSA through various methods, depicted diagrammatically.

| 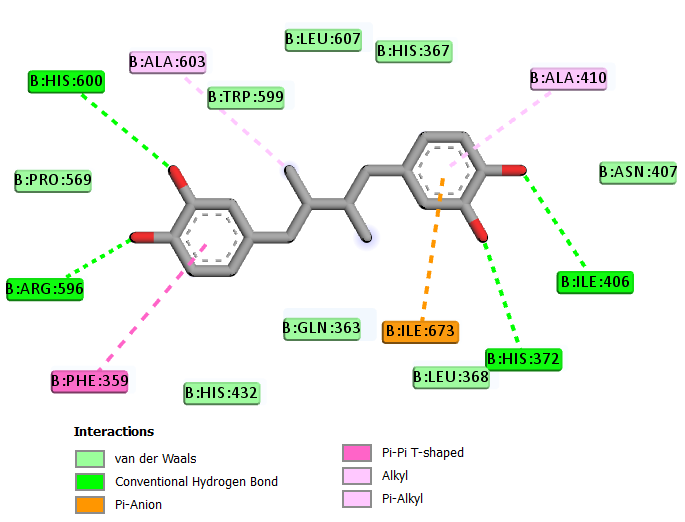 | 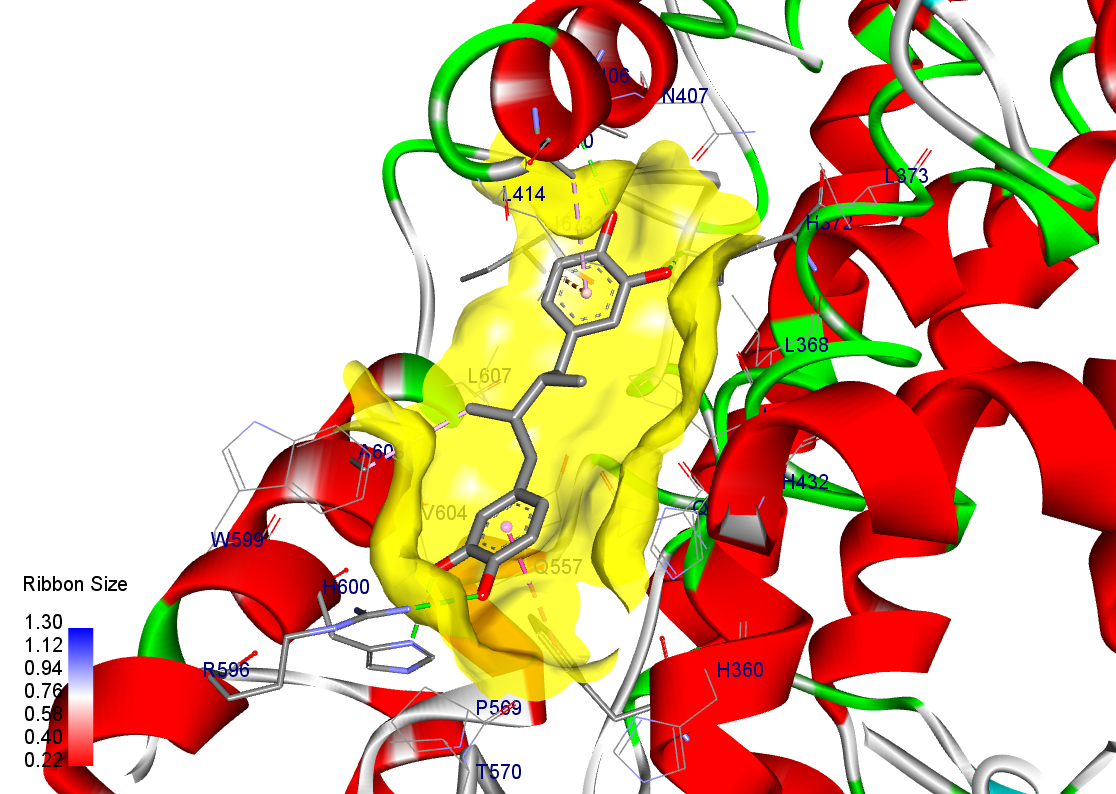 |
| --- | --- |

**Figure S2**: 2D (left) and 3D (right) Binding mode of co-crystal ligand (**NDGA**) in the active site of 5-LOX enzyme.

| 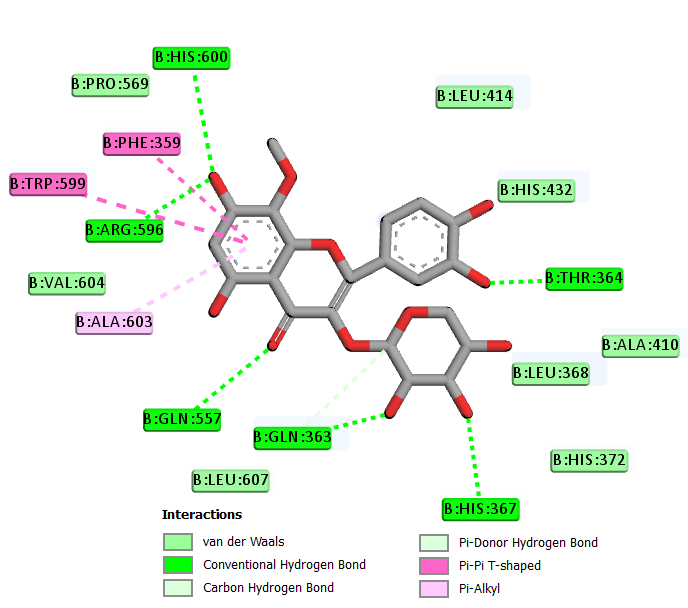 | 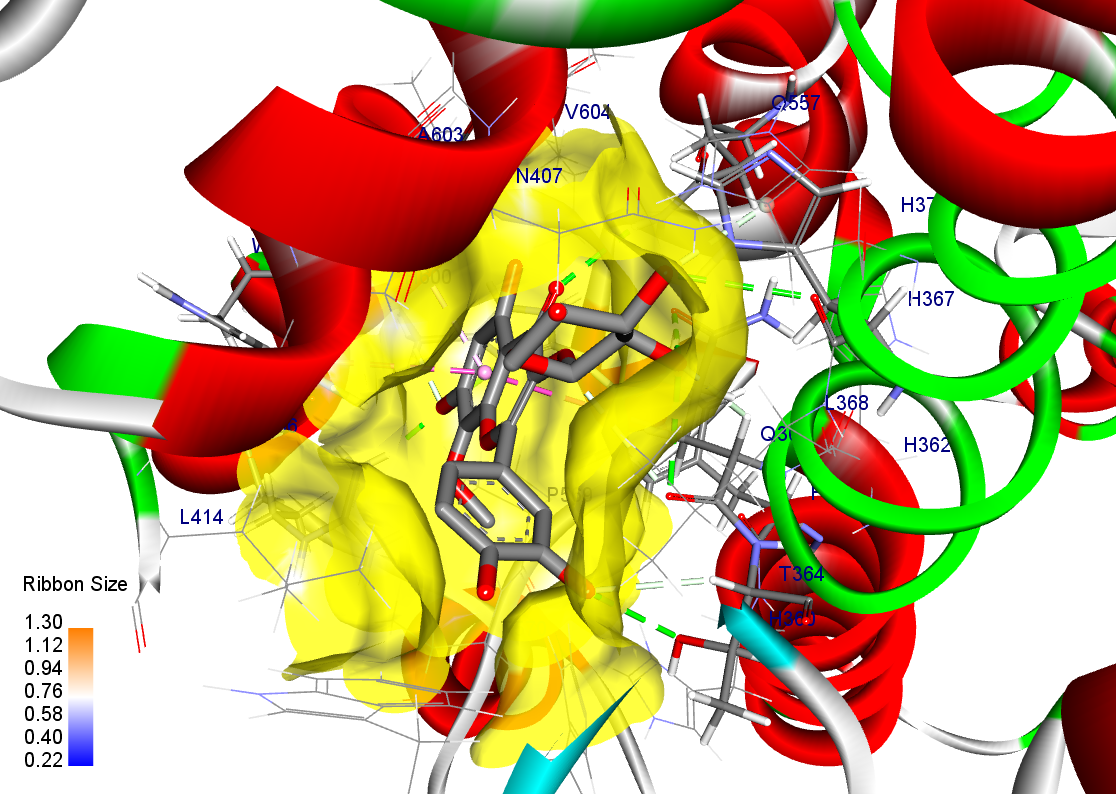 |
| --- | --- |

**Figure S3**: 2D (left) and 3D (right) Binding mode of **gossypetin 8-methoxy, 3-*O*-*β*-*D*-xylopyranoside** (metabolite 1) in the active site of 5-LOX enzyme.

| 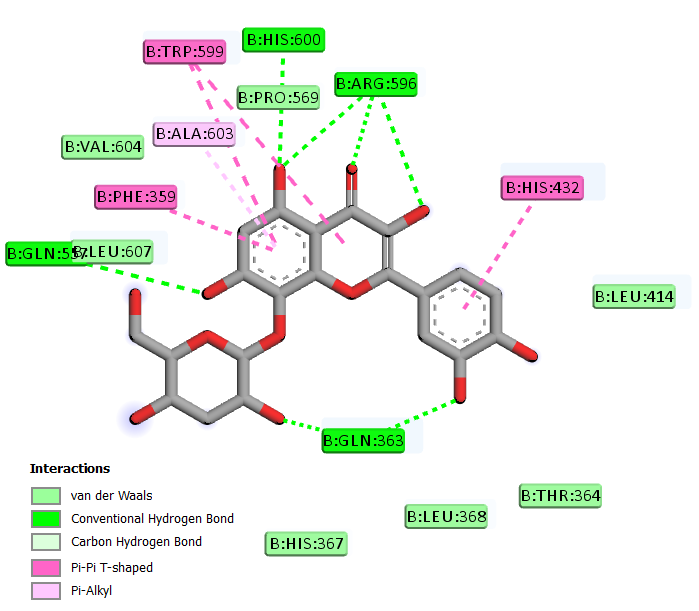 | 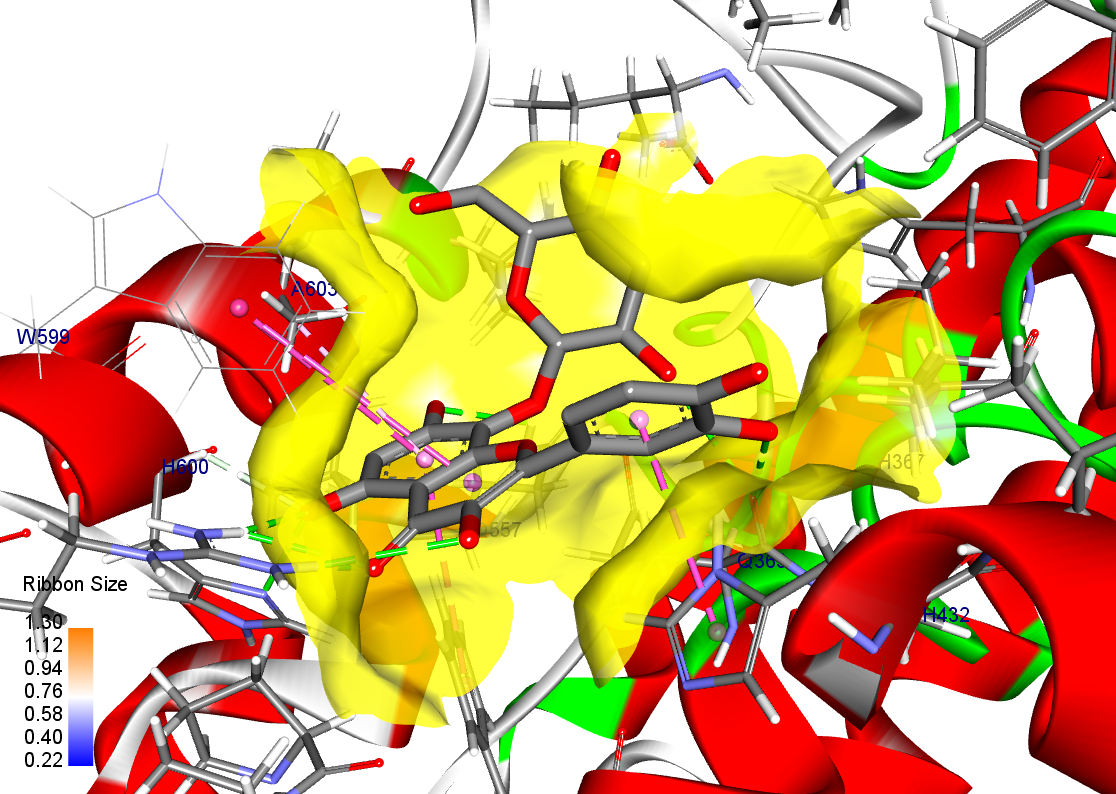 |
| --- | --- |

**Figure S4**: 2D (left) and 3D (right) Binding mode of **gossypetin 8-*O* *β*-*D* – glucopyranoside** (metabolite 2) in the active site of 5-LOX enzyme.

| 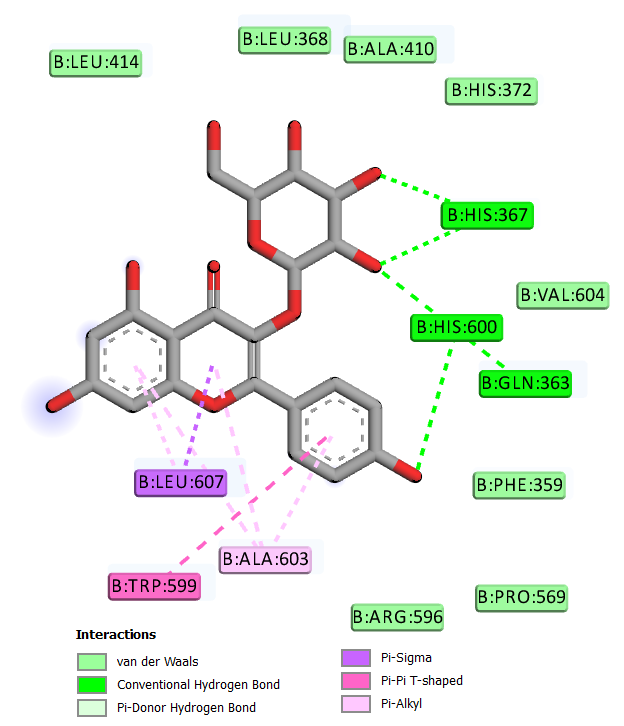 | 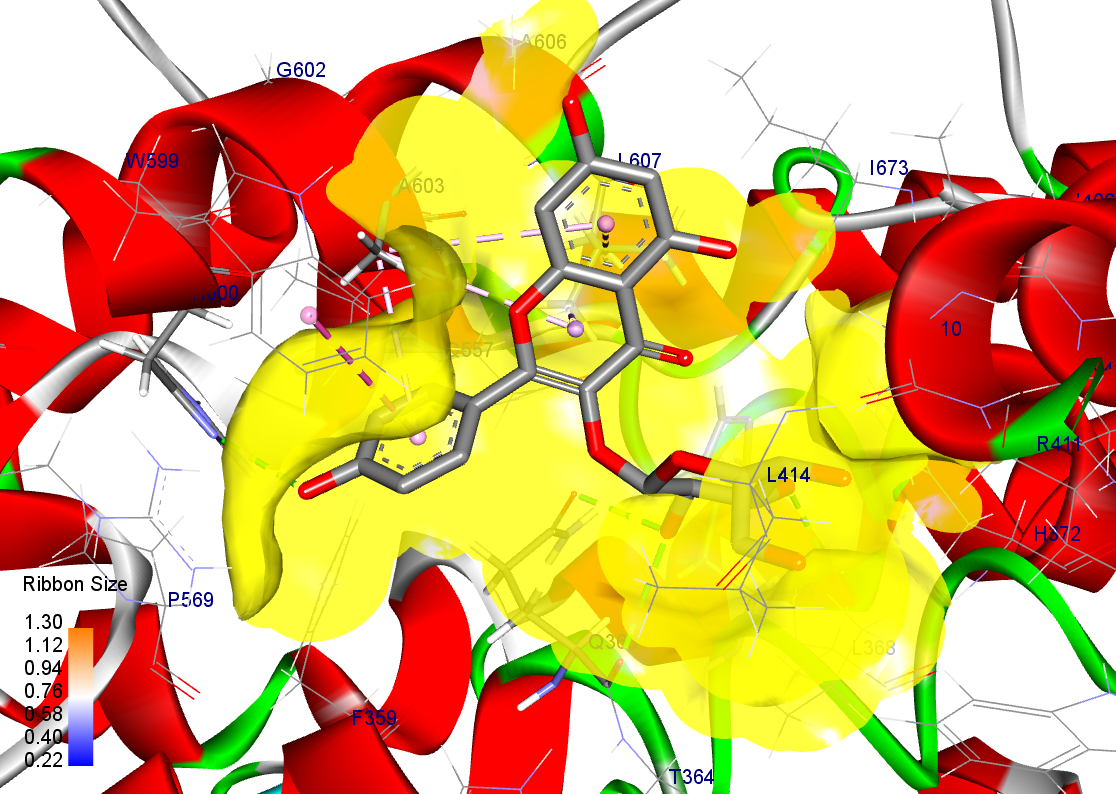 |
| --- | --- |

**Figure S5**: 2D (left) and 3D (right) Binding mode of **kaempferol 3-O*-ß–D*-glucoside** (metabolite 3) in the active site of 5-LOX enzyme.

| 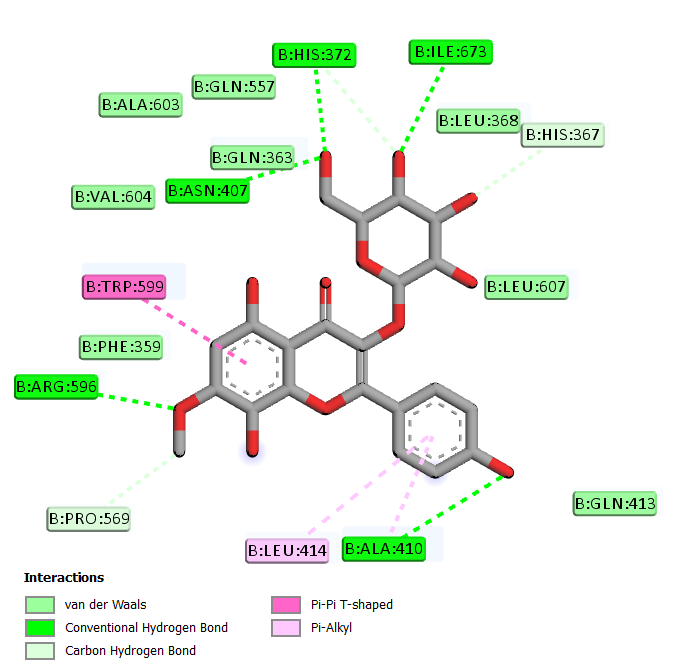 | 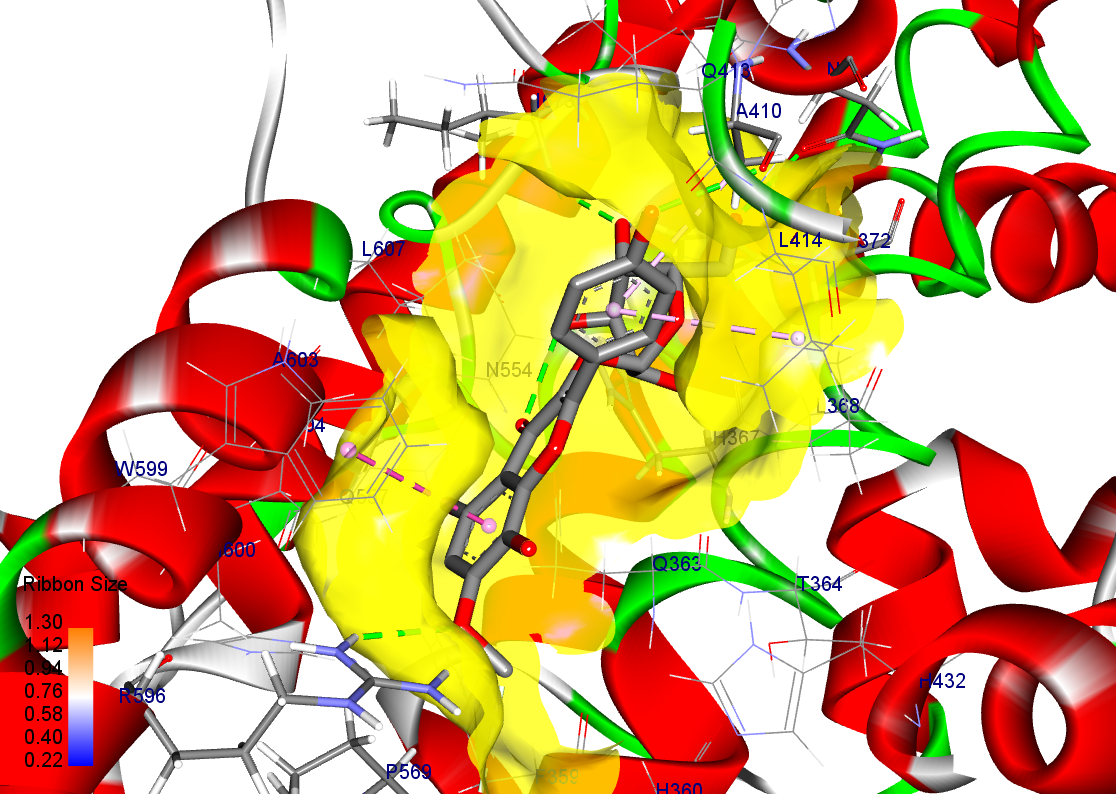 |
| --- | --- |

**Figure S6**: 2D (left) and 3D (right) Binding mode of **herbacetin-7-methyl ether-3-O*-β-D*-glucopyranoside** (metabolite 4) in the active site of 5-LOX enzyme.

| 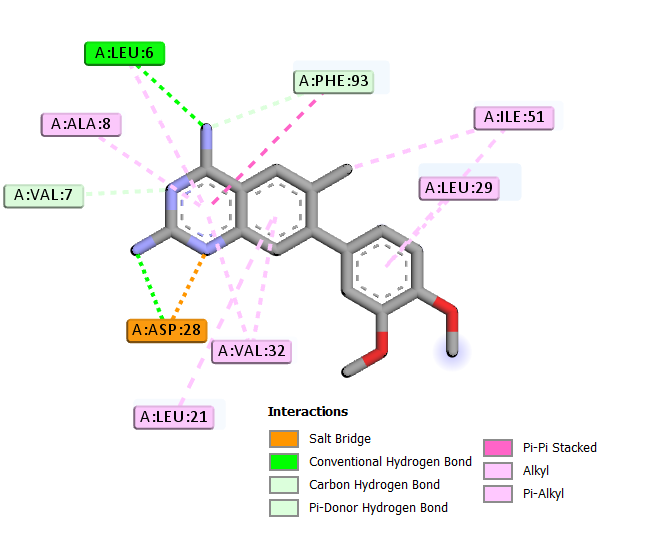 | 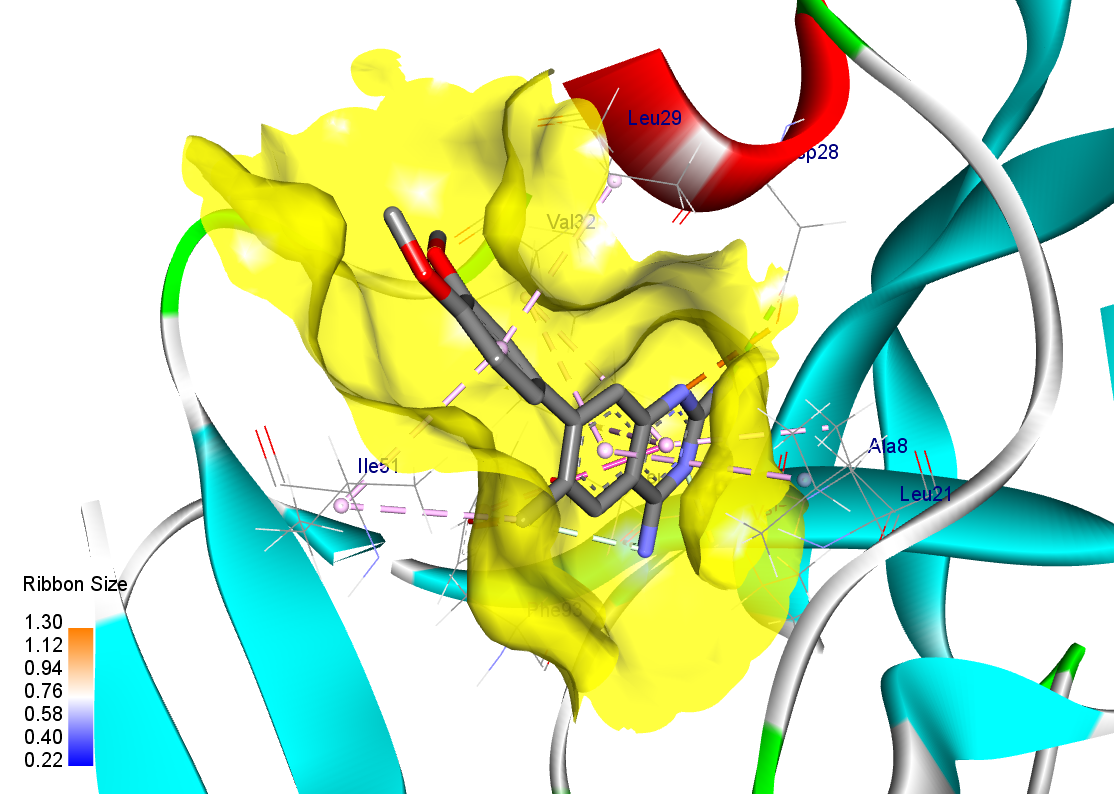 |
| --- | --- |

**Figure S7**: 2D (left) and 3D (right) Binding mode of **Q21** in the active site of DHFR enzyme.

| 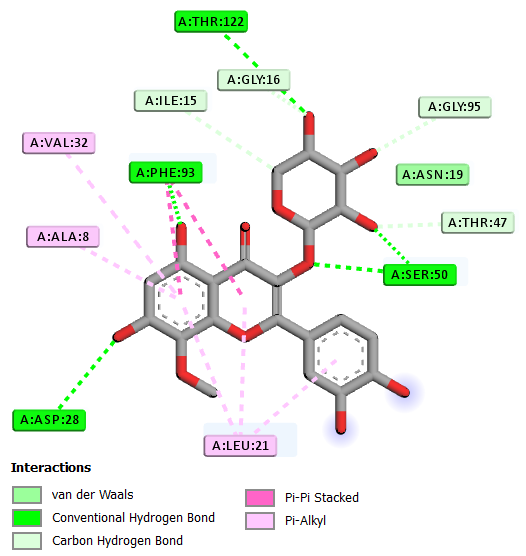 | 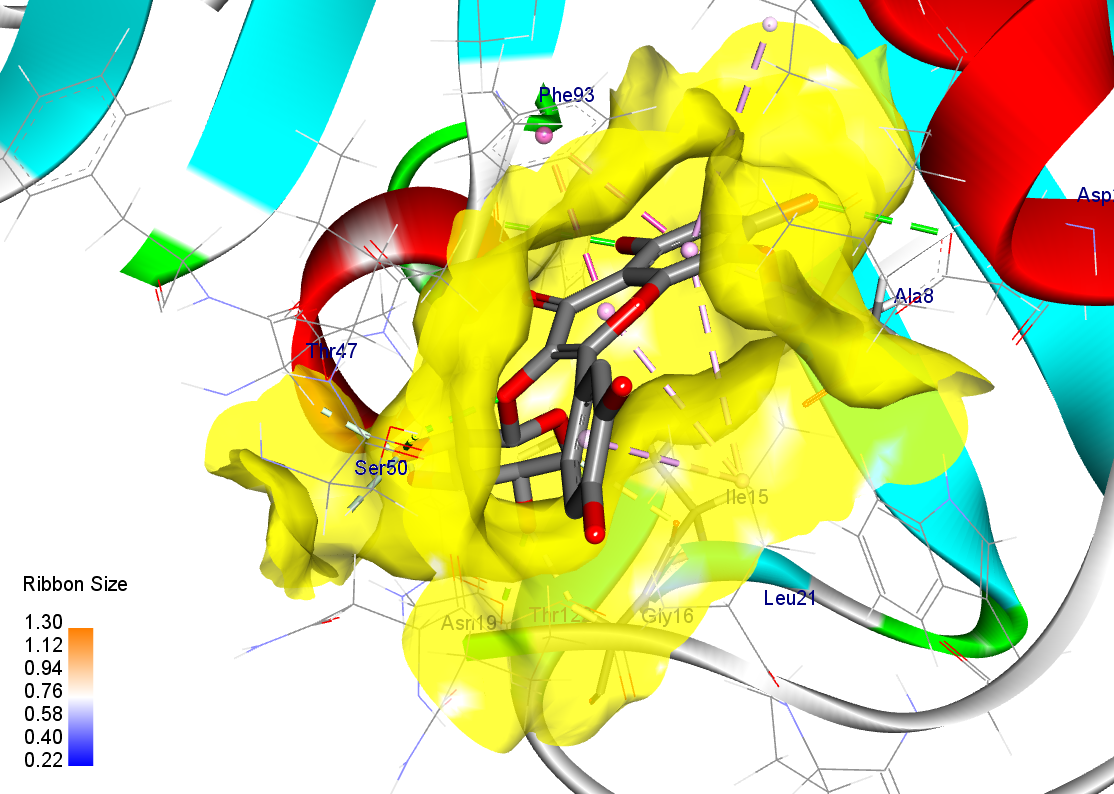 |
| --- | --- |

**Figure S8**: 2D (left) and 3D (right) Binding mode of **gossypetin 8-methoxy, 3-O-*β*-*D*-^4^C1-xylopyranoside** in the active site of DHFR enzyme.

| 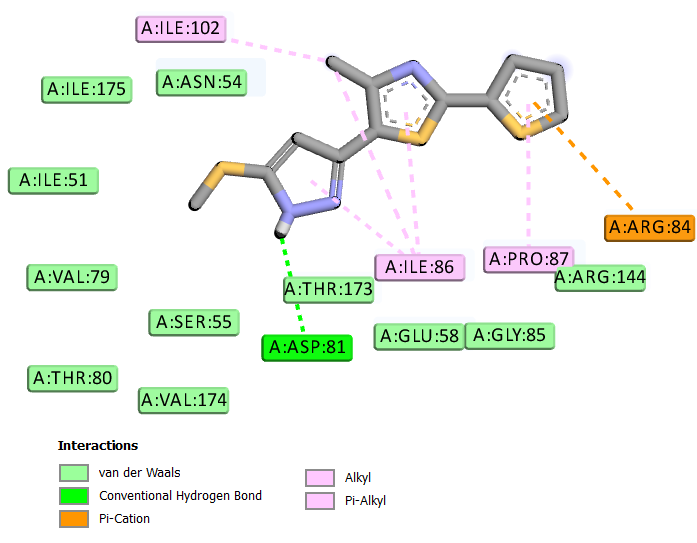 | 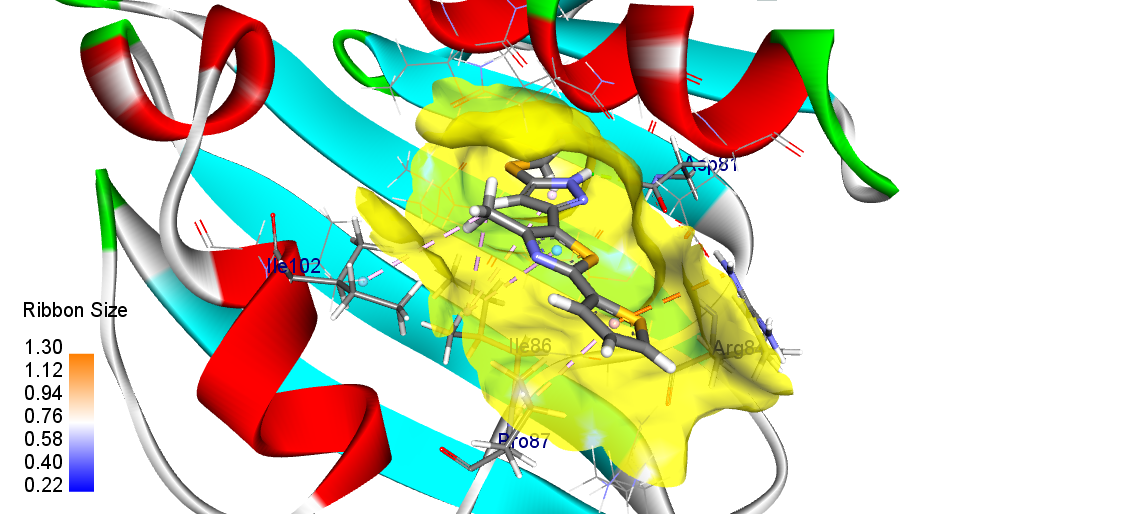 |
| --- | --- |

**Figure S9**: 2D (left) and 3D (right) Binding mode of co-crystal ligand ([B48](https://www.rcsb.org/ligand/B48)) into the ATP binding site of DNA gyrase B enzyme.

| 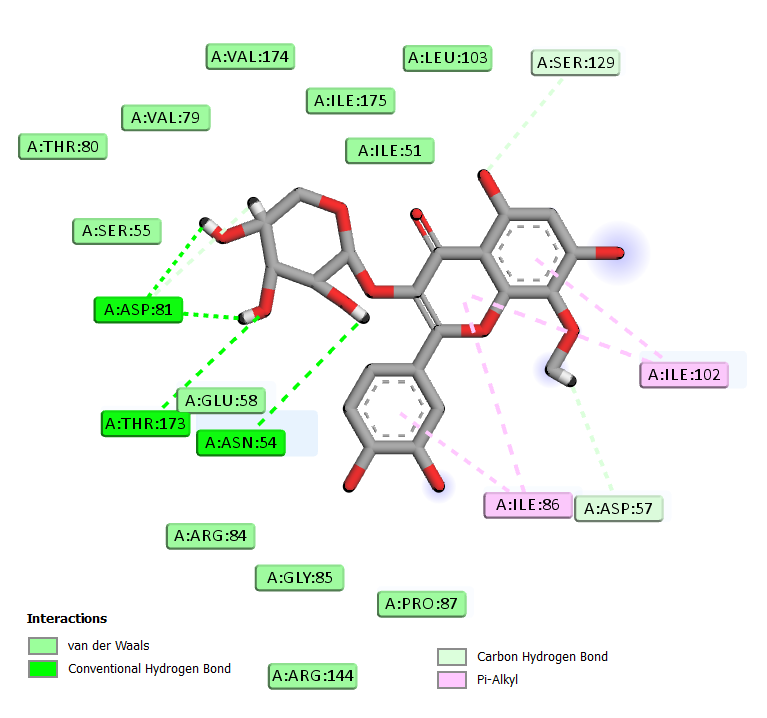 | 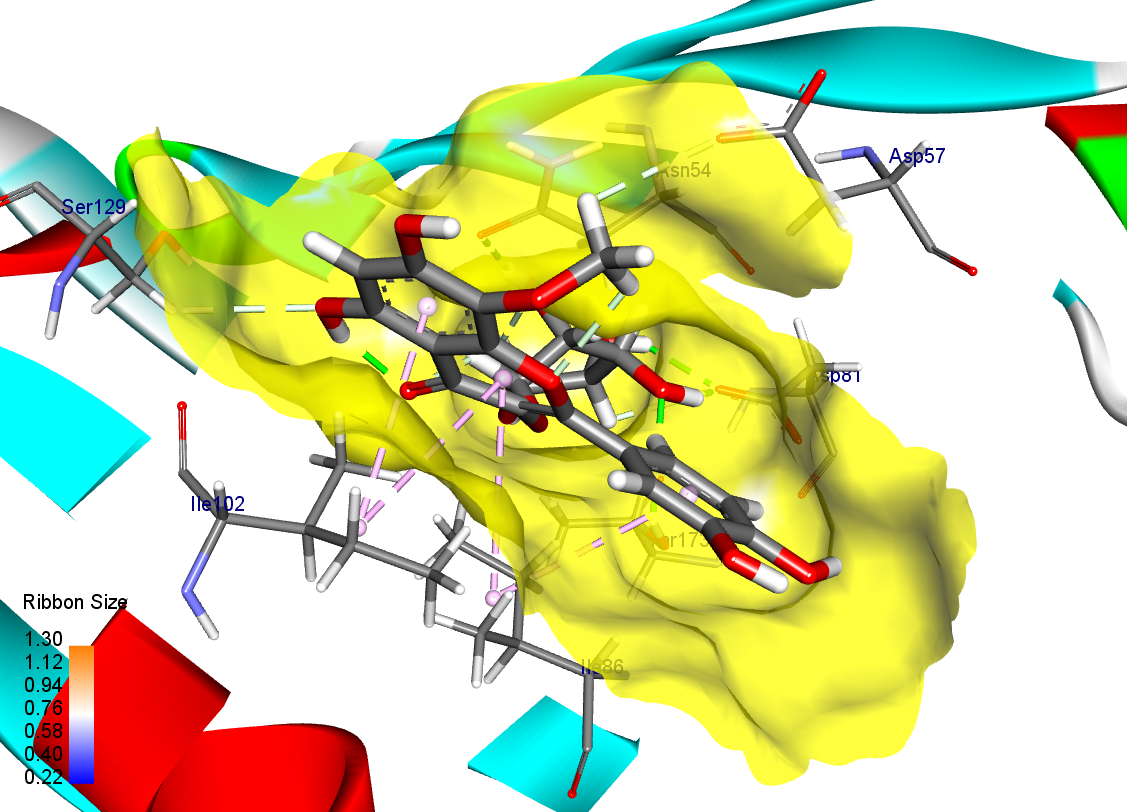 |
| --- | --- |

**Figure S10**: 2D (left) and 3D (right) Binding mode of **gossypetin 8-methoxy, 3-O-*β*-*D*-xylopyranoside** into the ATP binding site of DNA gyrase B enzyme.

| 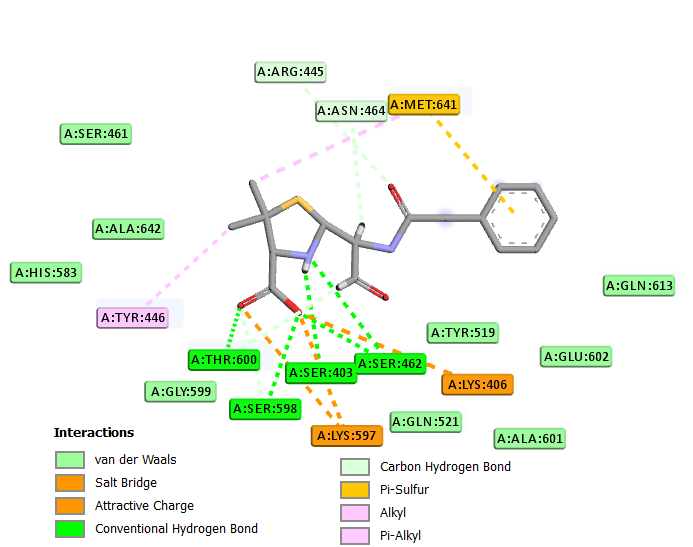 | 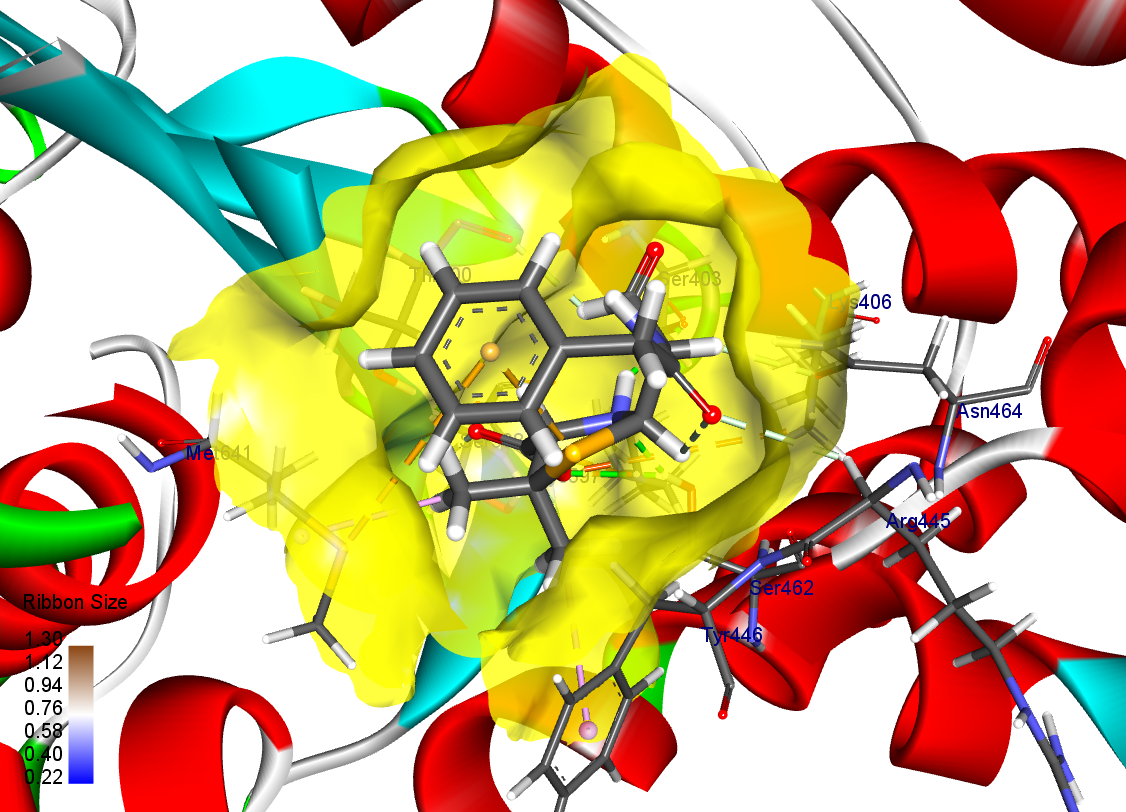 |
| --- | --- |

**Figure S11**: 2D (left) and 3D (right) Binding mode of co-crystal ligand ([PNM](https://www.rcsb.org/ligand/PNM)) into PBP2a enzyme's binding site.

| 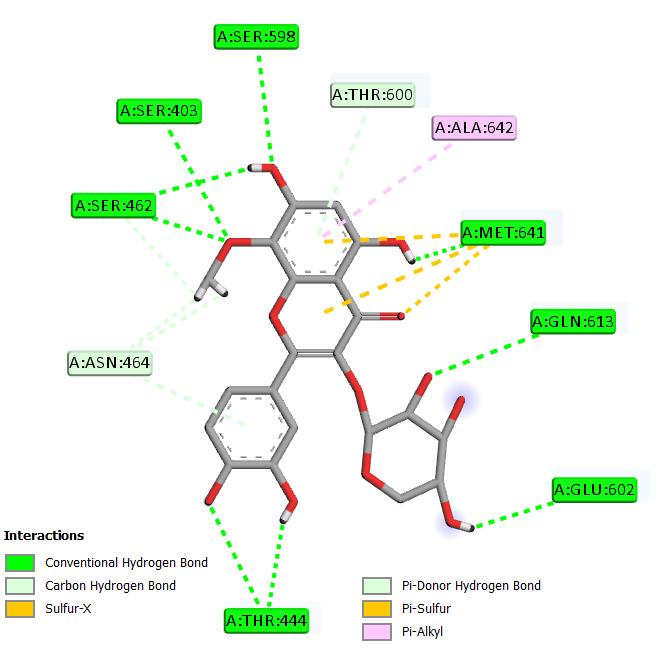 | 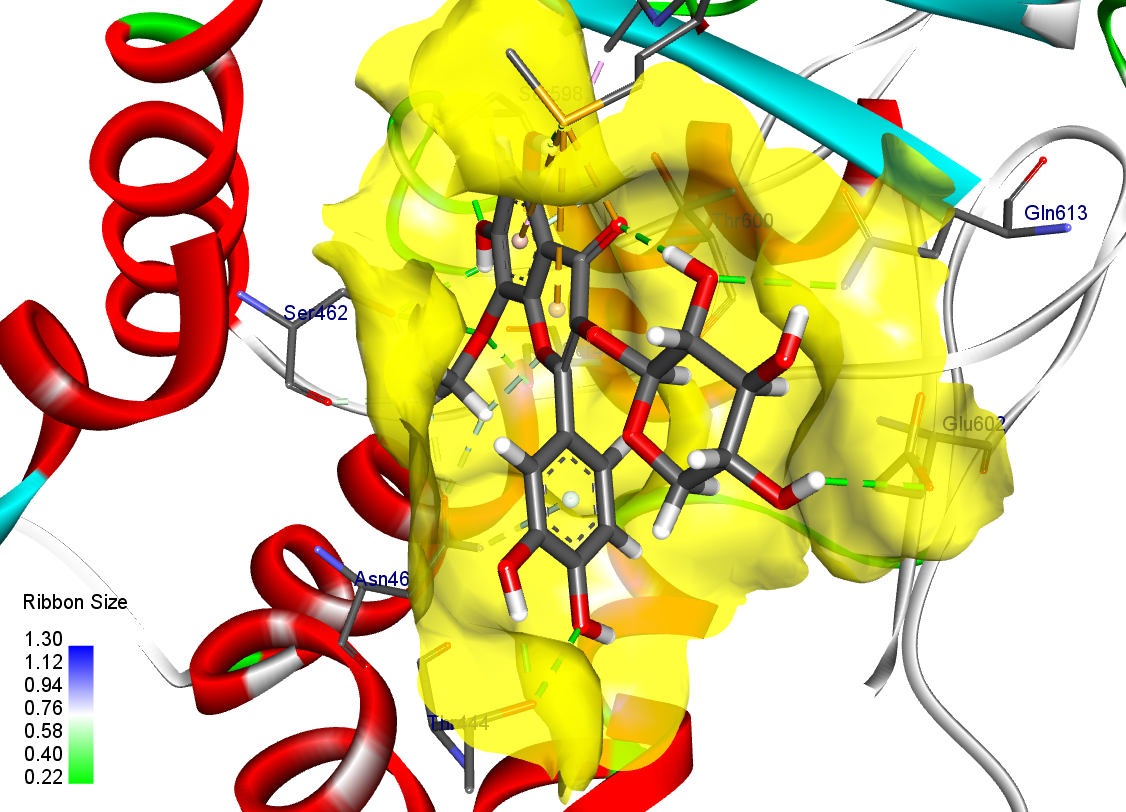 |
| --- | --- |

**Figure S12**: 2D (left) and 3D (right) Binding mode **gossypetin 8-methoxy, 3-O-*β*-*D*-^4^C1-xylopyranoside** into PBP2a enzyme's binding site.

| 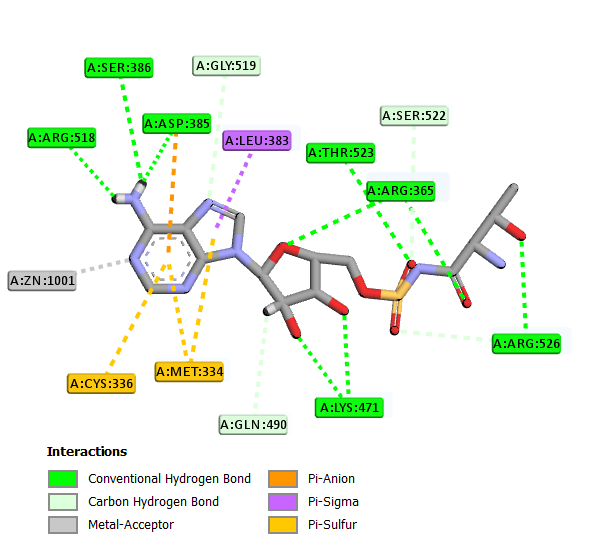 | 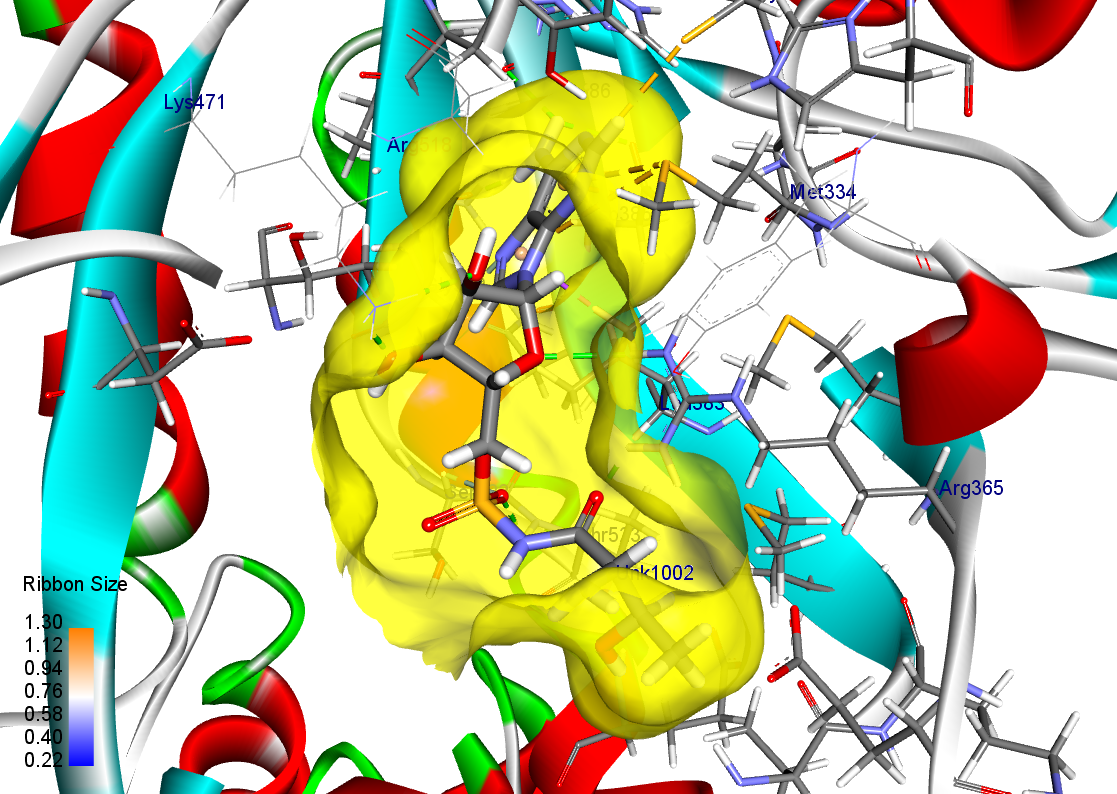 |
| --- | --- |

**Figure S13**: 2D (left) and 3D (right) Binding mode of co-crystal ligand into ThrRS enzyme's binding site.

| 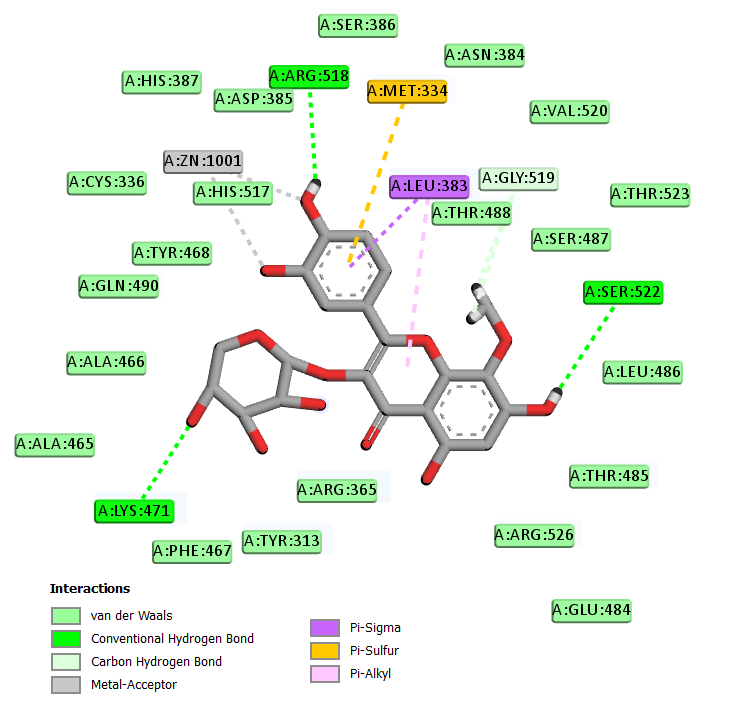 | 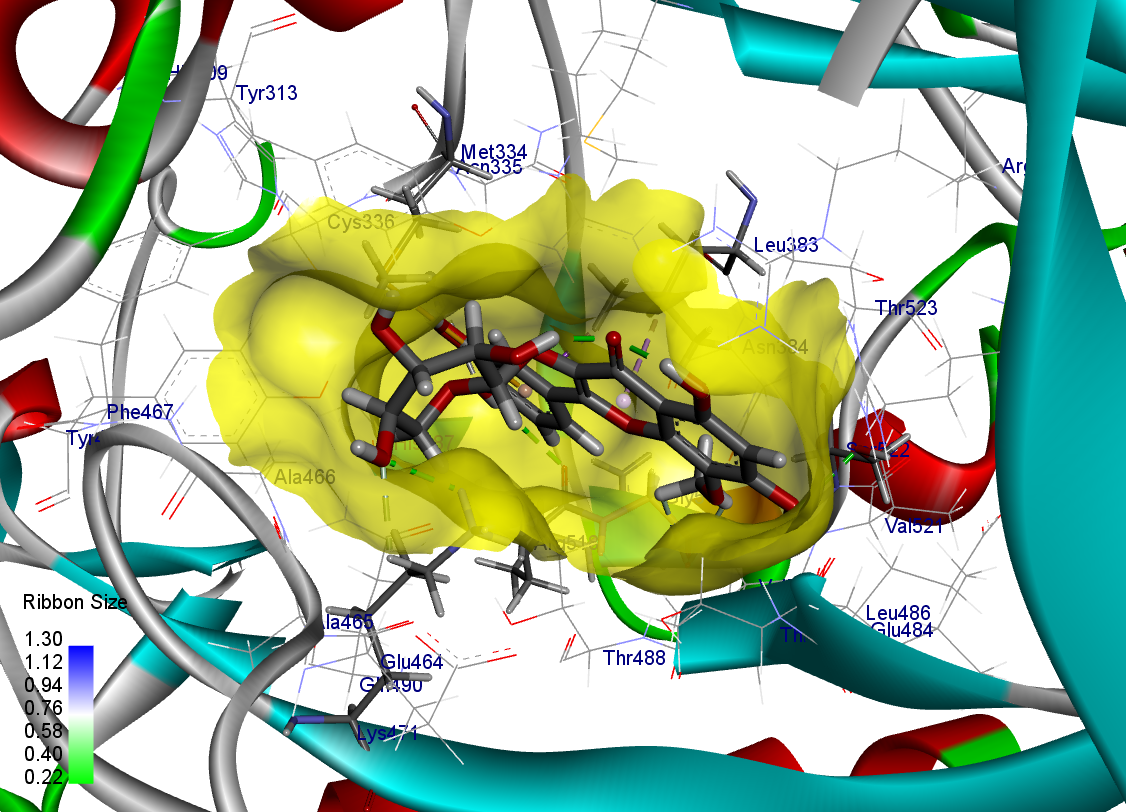 |
| --- | --- |

**Figure S14**: 2D (left) and 3D (right) Binding mode **gossypetin 8-methoxy 3-*O*-*β*-*D*-xylopyranoside** into ThrRS enzyme's binding site.


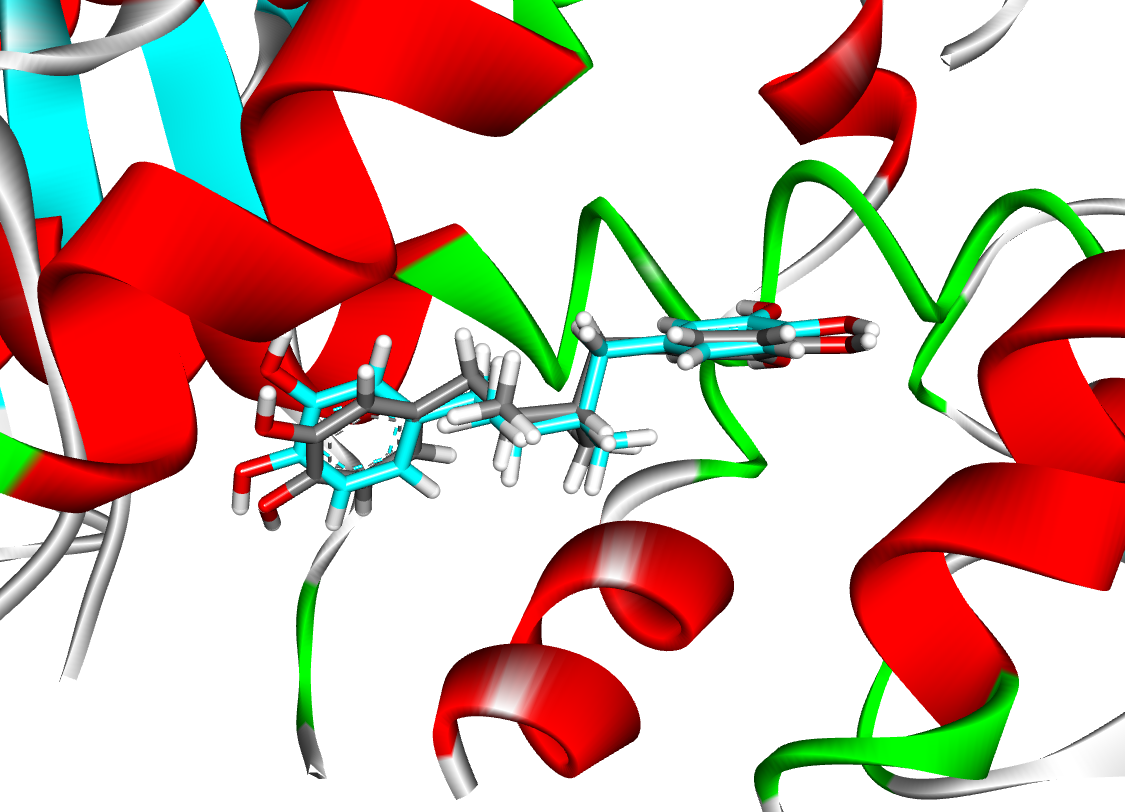


**Figure S15.** The root means square deviation between the original and docked poses of the cocrystal ligands of Lipoxygenase enzyme (PDB: 6n2w) was 0.62 Å.


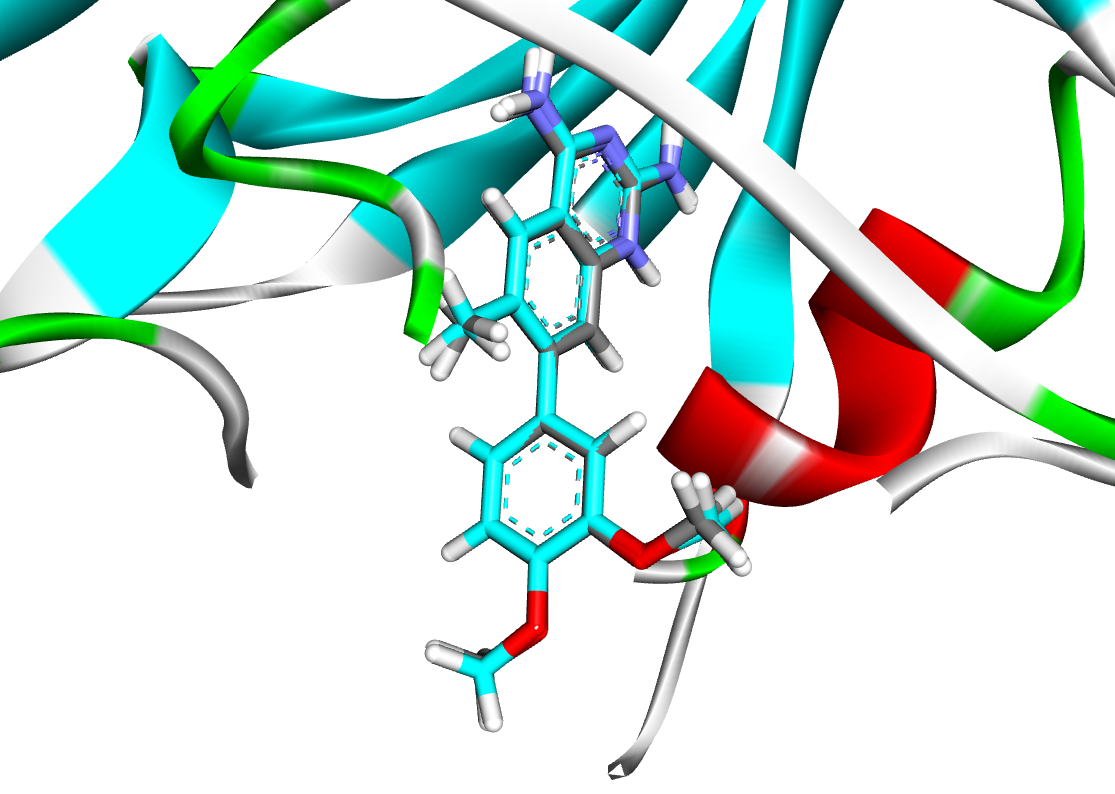


**Figure S16.** The root means square deviation between the original and docked poses of the cocrystal ligands of **dihydrofolate reductase** enzyme (PDB: 3sr5) was 0.12 Å.


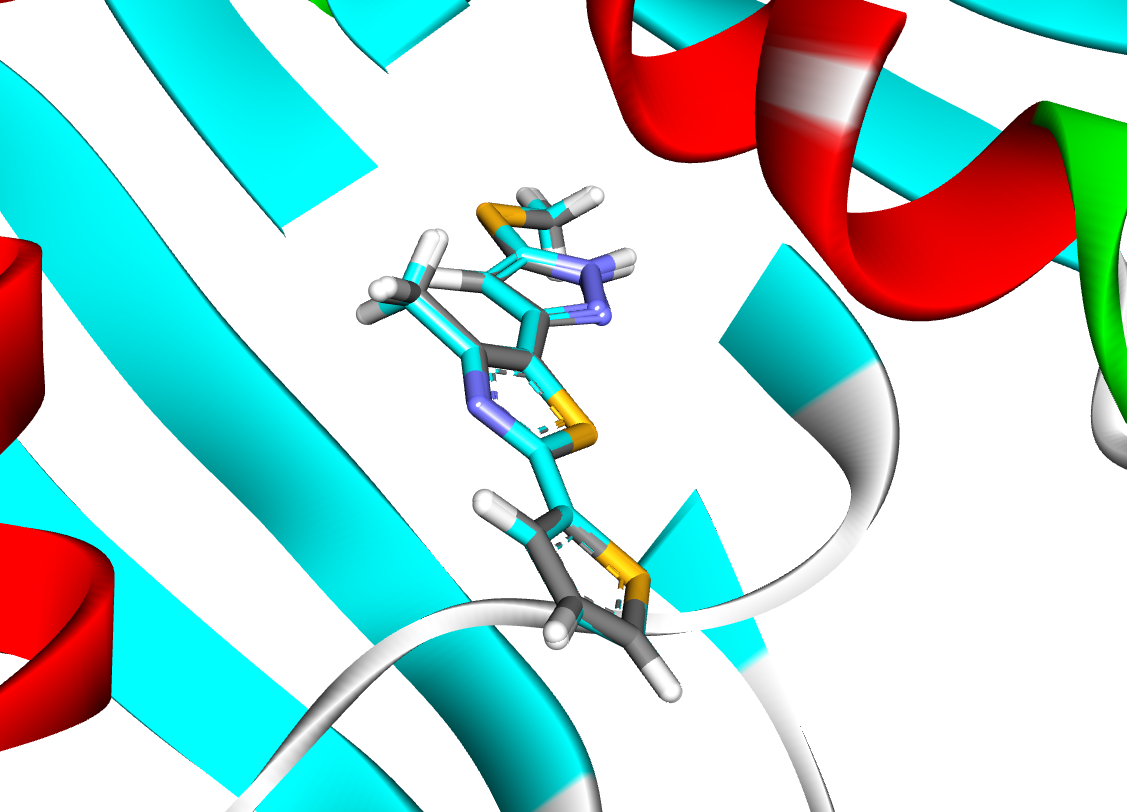


**Figure S17.** The root means square deviation between the original and docked poses of the cocrystal ligands of **DNA gyrase** enzyme (PDB: 3g75) was 0.08 Å.


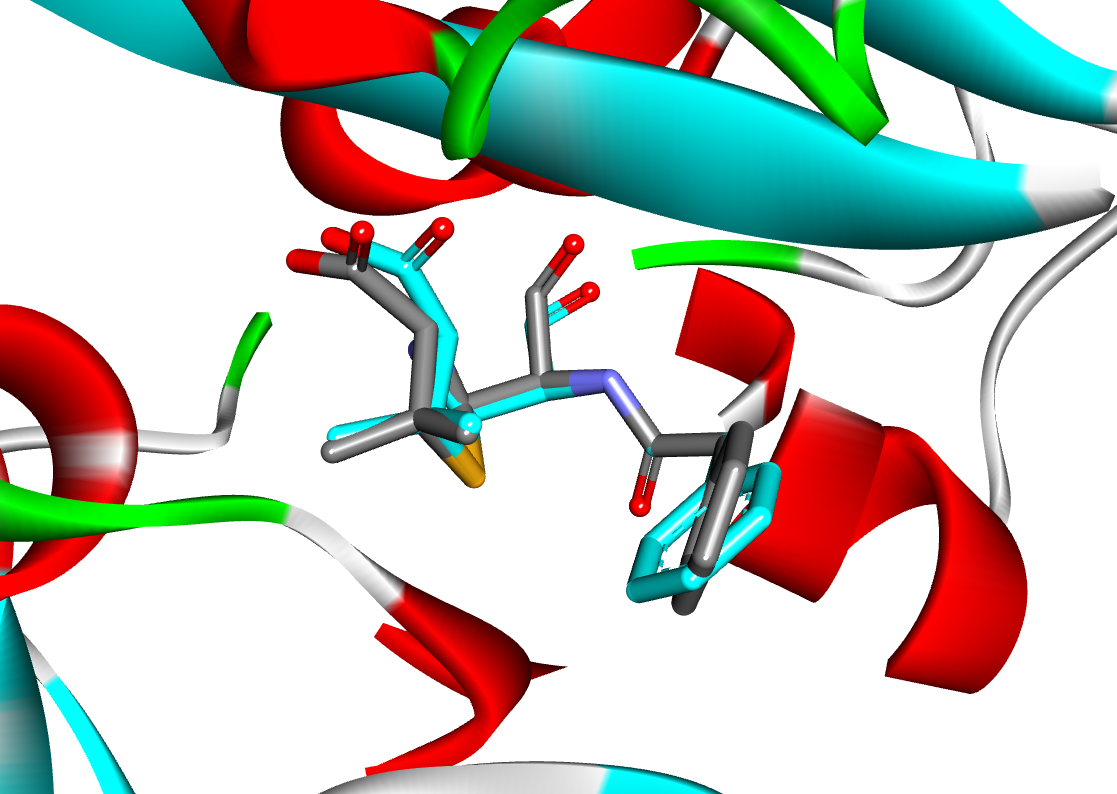


**Figure S18.** The root means square deviation between the original and docked poses of the cocrystal ligand ([PNM](https://www.rcsb.org/ligand/PNM)) of **Penicillin binding protein (PBP2a) enzyme (PDB: 1mwt)** was 0.70 Å.


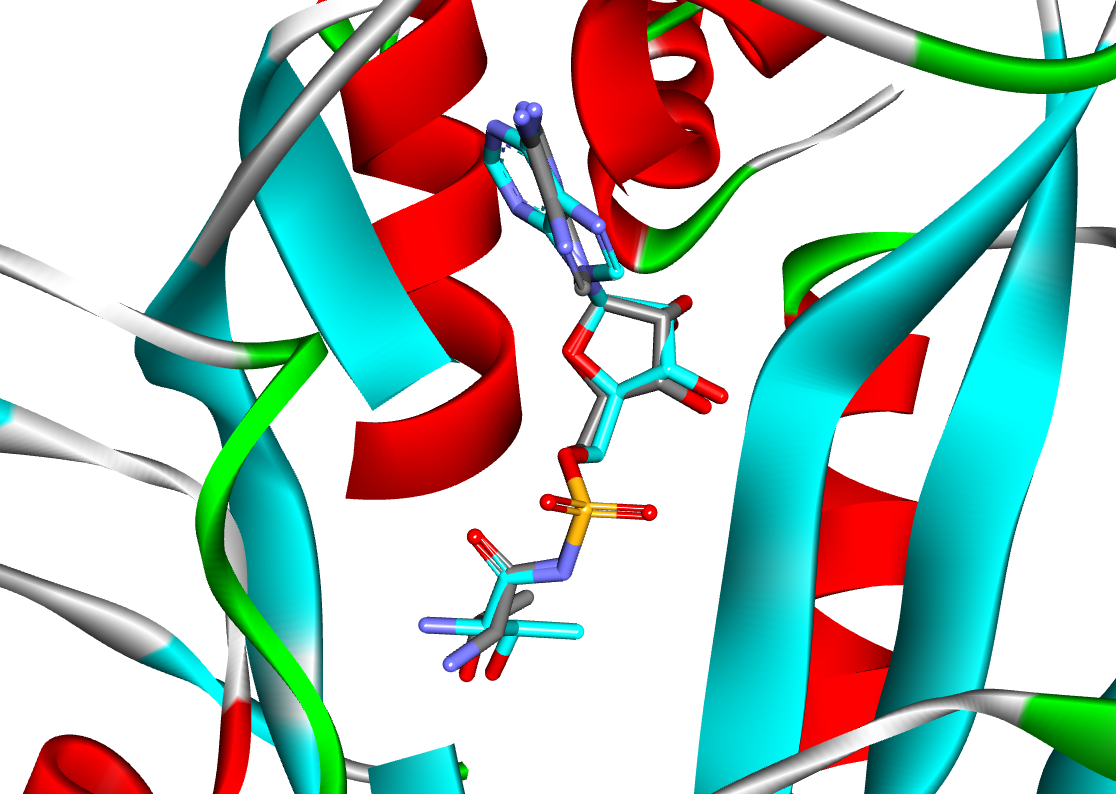


**Figure S19.** The root means square deviation between the original and docked poses of the cocrystal ligands of **threonyl-tRNA Synthetase (PDB: 1nyq)** was 0.65 Å.
